# A multifaceted crosstalk between brassinosteroid and gibberellin regulates the resistance of cucumber to *Phytophthora melonis*

**DOI:** 10.1111/tpj.16855

**Published:** 2024-06-03

**Authors:** Yunyan Kang, Zhongli Jiang, Chen Meng, Xianpeng Ning, Gengzheng Pan, Xian Yang, Min Zhong

**Affiliations:** ^1^ College of Horticulture South China Agricultural University Guangzhou P. R. China

**Keywords:** brassinosteroids, gibberellin, cucumber, hormone interactions, *Phytophthora melonis*

## Abstract

Cucumber plants are highly susceptible to the hemibiotroph oomycete *Phytophthora melonis*. However, the mechanism of resistance to cucumber blight remains poorly understood. Here, we demonstrated that cucumber plants with impairment in the biosynthesis of brassinosteroids (BRs) or gibberellins (GAs) were more susceptible to *P. melonis*. By contrast, increasing levels of endogenous BRs or exogenously application of 24‐epibrassinolide enhanced the resistance of cucumber plants against *P. melonis*. Furthermore, we found that both knockout and overexpression of the BR biosynthesis gene *CYP85A1* reduced the endogenous GA_3_ content compared with that of wild‐type plants under the condition of inoculation with *P. melonis*, and the enhancement of disease resistance conferred by BR was inhibited in plants with silencing of the GA biosynthetic gene *GA20ox1* or *KAO*. Together, these findings suggest that GA homeostasis is an essential factor mediating BRs‐induced disease resistance. Moreover, BZR6, a key regulator of BR signaling, was found to physically interact with GA20ox1, thereby suppressing its transcription. Silencing of *BZR6* promoted endogenous GA biosynthesis and compromised GA‐mediated resistance. These findings reveal multifaceted crosstalk between BR and GA in response to pathogen infection, which can provide a new approach for genetically controlling *P. melonis* damage in cucumber production.

## Significance Statement

This study demonstrates that GA homeostasis is necessary for the BRs‐mediated resistance of cucumber to *Phytophthora melonis*. BZR6, a key regulator of BR signaling, directly suppresses the transcription of *GA20ox1* and consequently reduces GA_1_ and GA_3_ contents. In conclusion, our results reveal the multifaceted regulation mechanism of BR/GA cooperation in growth–defense trade‐offs.

## INTRODUCTION

Phytophthora blight has been pronounced as a destructive disease of Solanaceae and Cucurbitaceae vegetable crops worldwide. Infection can occur at any plant stage, causing root, crown, and fruit rot, along with leaf blight (Reis et al., 2018; Wu et al., 2016). Phytophthora root and crown rot cause lesions on the roots and lower stems, leading to stem girdling, plant wilting, and ultimately plant death (Quesada‐Ocampo et al., 2023). Great efforts have been made to screen resistant lines or resistance‐related genes in Cucurbitaceae (Ayala‐Donas et al., 2022; Donahoo et al., 2013; Hashemi et al., 2020; Kousik et al., 2021; LaPlant et al., 2020; Wu et al., 2014), and to develop molecular markers based on quantitative trait loci associated with resistance to root and crown rot (Ramos et al., 2020; Vogel et al., 2021). However, the markers identified to date have not been successfully bred into cultivars with marketable standards, and the poor horticultural performance of resistant lines has limited their application in production. Therefore, overcoming the damage caused by root and crown rot disease remains the primary focus of breeding efforts in Cucurbitaceae production.

Brassinosteroids (BRs) are a group of polyhydroxylated plant steroid hormones that play vital roles in plant growth and development (Li et al., 2020; Nolan et al., 2020). Brassinolide (BL) and its immediate precursor castasterone (CS) are two of the most biologically active BRs identified in higher plants to date. The CYP85A in the cytochrome P450 family catalyzes the C‐6 oxidation of 6‐deoxocastasterone to CS and the subsequent conversion of CS to BL, which plays a crucial role in the regulation of biologically active BR levels (Kwon & Choe, 2005). Binding of active BR to the receptor brassinosteroid‐insensitive 1 (BRI1) triggers a series of transduction events, eventually leading to the dephosphorylation and activation of Brassinozole Resistant 1/bri1 EMS‐Suppressor 1 (BZR1/BES1). Finally, the dephosphorylated BZR1/BES1 regulates the expression of BR target genes to elicit specific physiological processes (Clouse, 2011).

Beyond their primary functions in the plant growth and development, BRs are also involved in growth‐defense trade‐offs. The BR signaling pathway exhibits cross‐talk with various immune‐related pathways triggered by diverse microbe‐ and pathogen‐associated molecular patterns at the receptor, cytoplasmic, or transcriptional level (Antolín‐Llovera et al., 2012; Bar et al., 2010; Bjornson et al., 2016; Chen & Shimamoto, 2011; Feng et al., 2021; Kalachova et al., 2022; Lin et al., 2014; Ranf et al., 2012; Saini et al., 2022; Turnbull et al., 2019; Yang et al., 2007; Yu et al., 2018; Zhang & Zhou, 2010). In *Nicotiana benthamiana*–*Phytophthora sojae* interactions, the virulence effector aldose 1‐epimerase (AEP1) activates plant immunity through mediating *P. sojae* extracellular sugar uptake, which depends on the BR receptor brassinosteroid insensitive 1‐associated receptor kinase 1 (BAK1) (Xu et al., 2021). AVR2, an RXLR effector secreted by the potato blight pathogen *Phytophthora infestans*, suppresses cell death triggered by the elicitin Infestin 1 through activating the expression of the BR‐responsive marker gene *StCHL1* (Turnbull et al., 2017). The cytoplasmic fraction of BZR1 was also confirmed to positively regulate effector‐triggered immunity mediated by the resistance protein RPS4 in the TIR‐NB‐LRR protein class; however, this effect was weakened with the nuclear translocation of BZR1 (Qi et al., 2021). In addition, the tomato BR‐insensitive mutant *curl3* exhibited reduced disease resistance against *P. infestans* (Dallagnol et al., 2023). Overexpressing *BES1* could enhance the resistance of apple to *Valsa mali* attack (Liu et al., 2021). Our previous study also confirmed the suppressive effect of 24‐epibrassinolide (EBR) on *Phytophthora melonis* infection through regulating the development of the hypocotyl and vascular bundle in cucumber plants (Ren et al., 2020).

BRs and gibberellins (GAs) are two important phytohormones that have similar effects on several processes of plant growth and development. BR‐ and GA‐deficient mutants show similar phenotypes to varied degrees, including decreased seed germination, de‐etiolation in the dark, dwarfism, and delayed flowering. The coordination and integration of BR and GA have been widely documented to play key roles in regulating plant growth, development, and photomorphogenesis. The two most common models of these interactions are the “signaling” model positing a DELLA‐BZR1 interaction (Ross & Quittenden, 2016) and the GA synthesis theory (Jager et al., 2005; Unterholzner et al., 2015). However, the mechanism by which interactions between BRs and GAs mediate the trade‐off between growth and defense in response to *P. melonis* infection in cucumber remains unclear. Here, we provide evidence that BR signaling fine‐tunes GA biosynthesis via BZR‐mediated transcriptional regulation of the key GA biosynthesis gene *gibberellin 20 oxidase 1* (*GA20ox1*) and that BR‐induced disease resistance is dependent on the interaction with GAs.

## RESULTS

### BR mediates the resistance of cucumber to *P. melonis*

**Figure 1:**
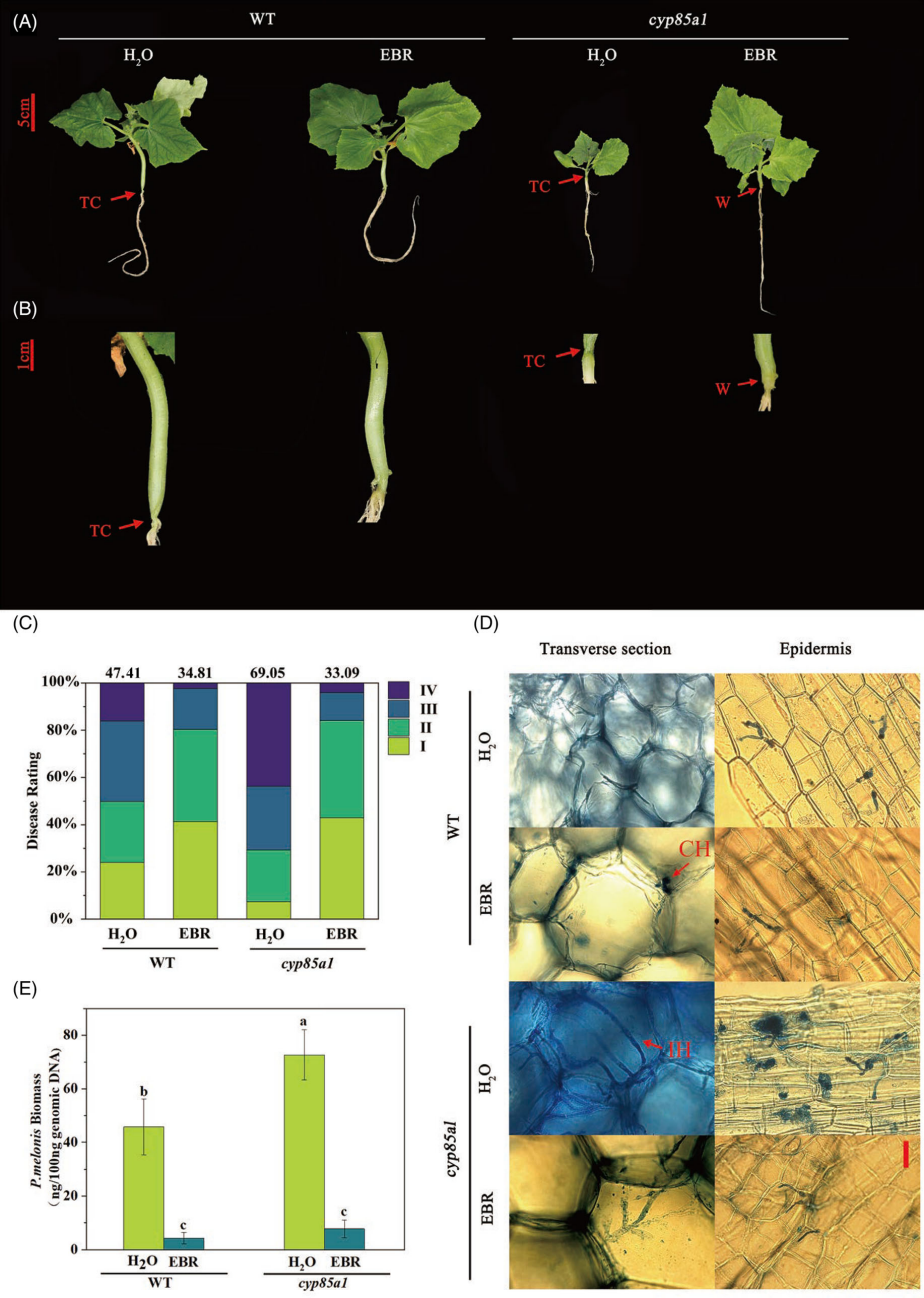
BRs suppressed *Phytophthora melonis*‐induced disease spreading on cucumber plants 3 days after inoculation. (A) Phenotypes of *P. melonis* infected *cyp85a1* and WT plants with or without EBR application. (B) Closeup of visible water‐soaking, brownish lesions, or plant collapse. (C) Disease incidence in the plants with and without EBR treatment infected with *P. melonis*. (D) Invasive hyphae extended on the epidermis and transverse section of hypocotyls stained with lactophenol‐trypan blue. (E) *P. melonis* biomass accumulation in the hypocotyls infected with *P. melonis*. CH, confined hypha; EBR, 24‐epibrassinolide; IH, invasive hypha; TC, tissue collapse; W, water soaking with necrosis; WT, wild type. Bars = 10 μm. Data in (E) are shown as means ± SD of three biological replicates (*n* = 9). Different letters indicated significant differences (*P* < 0.05) according to Duncan's multiple range tests.

To clarify the role of BRs in governing plant resistance to *P*. *melonis*, we first characterized the responses of WT and *cyp85a1* mutant plants, with and without exogenous EBR treatment, to inoculation with *P*. *melonis*. At 3 days after inoculation, seedlings of the *cyp85a1* mutant were severely susceptible to *P*. *melonis*, with plants exhibiting obvious shrinkage and water soaking with necrotic spots (Figure 1A,B); the disease index reached 69.05% (Figure 1C). However, WT plants displayed only mild disease symptoms with a disease index of 47.41%. Exogenous foliar application of 100 μM EBR was performed to test the potential infection‐suppression effect of EBR. As expected, supplying plants with EBR increased the resistance of cucumber plants to *P*. *melonis* infection as the disease index decreased to 34.81% and 33.09% in *cyp85a1* and WT plants, respectively, after EBR treatment (Figure 1C).

A histological analysis was performed to observe the infection process of *P*. *melonis*. At 3 days after inoculation, extensively branched hyphae became evident on the cross‐section and epidermal cells of the hypocotyls of *cyp85a1* plants (Figure 1D). However, only a few invasive and confined hyphae developed in untreated WT plants and in both EBR‐treated WT and *cyp85a1* plants. RT‐qPCR confirmed that the pathogen biomass in *cyp85a1* mutant plants was significantly higher than that in WT plants. The amount of *P*. *melonis* DNA per 100 ng genomic DNA in the hypocotyl was 72.65 ng in *cyp85a1* plants and was only 45.77 ng in WT plants, with 4.33 and 7.74 ng *P*. *melonis* DNA/100 ng genomic DNA in EBR‐treated WT and *cyp85a1* plants, respectively (Figure 1E).

### BR‐mediated Phytophthora blight resistance is associated with GA biosynthesis

**Figure 2:**
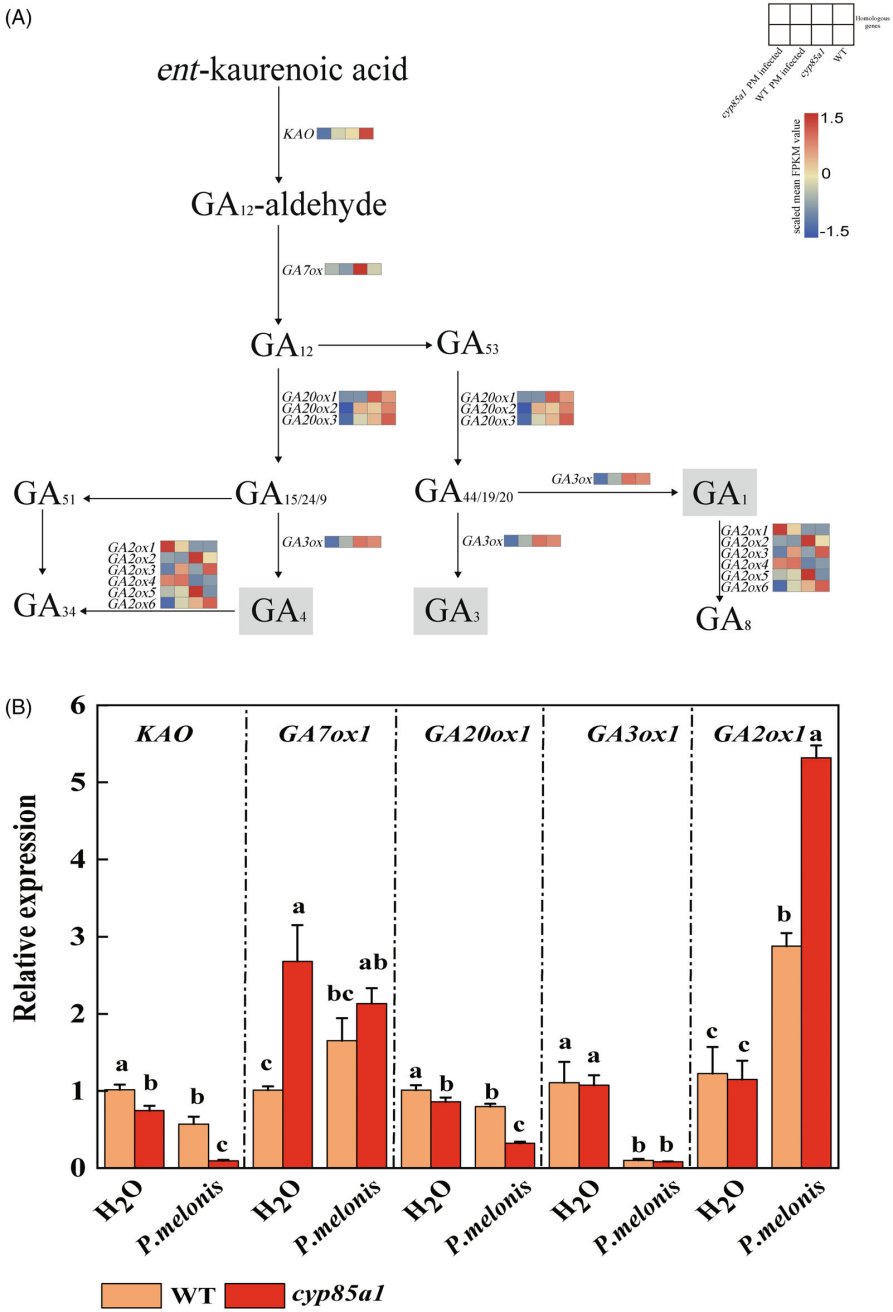
Transcriptional profiling of *Phytophthora melonis*‐infected hypocotyls of WT versus *cyp85a1* plants 3 days after inoculation revealed the majority of GA biosynthetic genes involved in BR‐mediated phytophthora blight resistance. (A) Loss of function of *CYP85A1* inhibited the expression of GA biosynthetic genes in *P. melonis*‐infected cucumber hypocotyls. Heatmap colors represent the gene expression (log_2_fold change) for each comparison (Tables S4 and S5). (B) Validation of RNA‐seq gene expression using quantitative real time PCR (qRT‐PCR). Data in (B) are shown as means ± SE of three biological replicates (*n* = 9). Different letters indicated significant differences (*P* < 0.05) according to Duncan's multiple range tests. *cyp85a1* PM infected, *P. melonis*‐infected *cyp85a1*; WT PM infected, *P. melonis*‐infected WT; WT, non‐inoculated WT; *cyp85a1*, non‐inoculated *cyp85a1*; WT, wild type.

To further explore the regulatory mechanism of BR‐mediated Phytophthora blight resistance, the hypocotyls of *cyp85a1* and WT plants with or without *P*. *melonis* infection were sampled at two‐leaf stage for comparative RNA‐sequencing analysis 3 days after *P. melonis* inoculation (Figure S2; Table S3). Based on global transcription changes, the expression of bioactive GA biosynthetic genes was found to be significantly hindered in the *cyp85a1* mutant upon *P*. *melonis* infection (Figure S3; Tables S4 and S5). The expression of *GA20ox3* (CsaV3_6G005990) and *gibberellin 3‐beta‐dioxygenase 1* (*GA3ox1*; CsaV3_7G032870) was all down‐regulated in response to *P*. *melonis* infection in *cyp85a1* mutant hypocotyls; however, the expression levels of these two genes did not significantly change in infected‐hypocotyls of WT plants. Consequently, the higher expression levels of *GA20ox3* and *GA3ox1* were observed in infected hypocotyls of WT plants as compared to those of *cyp85a1* mutant plants (Figure 2A, WT PM‐infected vs *cyp85a1* PM‐infected; Table S5). The expression of *KAO* (CsaV3_3G049400) was down‐regulated in infected‐hypocotyls of both genotypes upon infection but to a greater extent in *cyp85a1* mutant plants. The expression levels of *gibberellin 7 oxidase 1* (*GA7ox1*; CsaV3_1G005350) and *GA20ox1* (CsaV3_5G005560) decreased in infected‐hypocotyls of both genotypes upon infection to a similar extent. Moreover, the expression of *gibberellin 2 oxidase 1* (*GA2ox1*; CsaV3_4G007790), which encodes the enzyme that converts bioactive GA_4_ to inactive GA_34_, was up‐regulated in infected hypocotyls of both genotypes upon infection, but to a greater level in *cyp85a1* mutants than in WT plants. The *CYP85A1* mutation did not affect the expression of GA biosynthetic genes in non‐inoculated hypocotyls (Figure 2A, WT vs *cyp85a1*; Table S5). The expression patterns of the above DEGs were verified using RT‐qPCR (Figure 2B).

**Figure 3:**
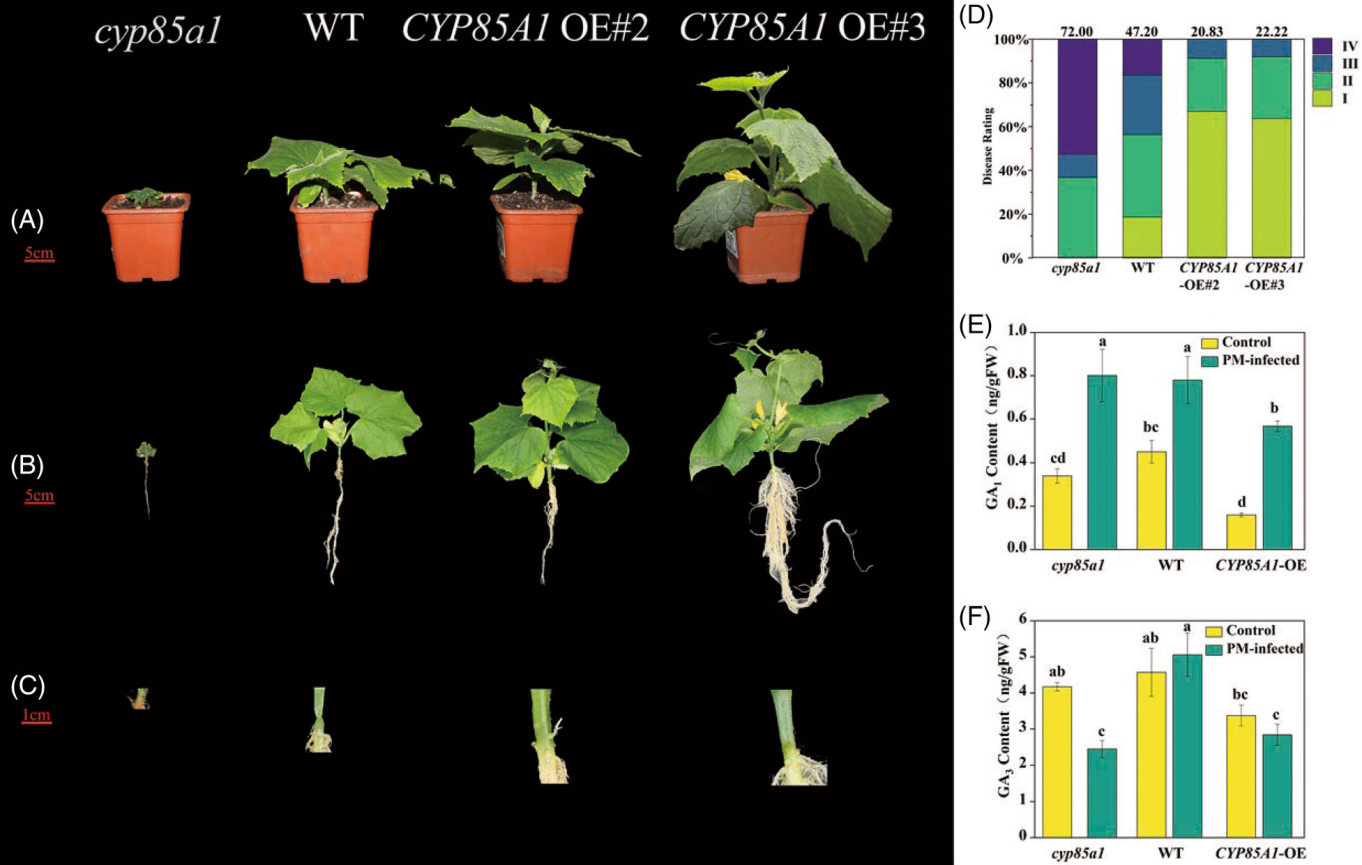
BR‐mediated phytophthora blight resistance is associated with GA biosynthesis. (A) Phenotypes of *cyp85a1*, WT and *CYP85A1*‐OE plants on day 0 before inoculation. (B) Phenotypes of *Phytophthora melonis* infected *cyp85a1*, WT and *CYP85A*1‐OE plants 3 days after inoculation. (C) Closeup of visible water‐soaking, brownish lesions, or plant collapse. (D) Disease incidence in *cyp85a1*, WT and *CYP85A1*‐OE plants. (E) Endogenous GA_1_ levels. (F) Endogenous GA_3_ levels. Data in (E, F) are shown as means ± SE of three biological replicates (*n* = 9). Different letters indicated significant differences (*P* < 0.05) according to Duncan's multiple range tests. EBR, 24‐epibrassinolide; WT, wild type.

Next, to confirm the interaction between different BR levels in the plants and growth, disease resistance, and GA biosynthesis, we further transformed the inbred cucumber line CCMC with the construct *35S:CYP85A1* into the pBI121‐GFP vector (Figure S4). Compared to those of the WT CCMC plants, *CYP85A1*‐OE lines exhibited an enhanced growth rate and improved disease resistance (Figure 3A–C). In addition, the hypocotyl thickness, hypocotyl length, leaf number, and leaf area of *CYP85A1*‐OE lines were significantly increased in comparison to those of WT plants (Figure 3A; Figure S5). Furthermore, the disease index of OE‐2 and OE‐3 plant lines decreased by 55.87% and 52.92% compared to that of the WT CCMC plants, whereas the EMS‐derived mutant *cyp85a1* deficient in BR biosynthesis showed a 52.54% increase in the disease index (Figure 3B–D). With respect to endogenous GAs biosynthesis, GA_1_ and GA_3_ levels significantly decreased in OE‐2 plants unpredictably, but there was no change in the levels of these hormones in the EMS‐derived mutant *cyp85a1* compared with those of WT plants under the non‐inoculation condition. However, after *P. melonis* inoculation, GA_1_ levels were increased in the *P. melonis*‐infected hypocotyls of all three genotypes compared to those of non‐inoculated plants, although the differences were not statistically significant. Notably, GA_3_ levels were markedly reduced in the *cyp85a1* mutant in response to pathogen infection, but did not change in either the WT or OE‐2 plants (Figure 3E,F). Thus, we speculated that the regulation of Phytophthora blight resistance by BRs might involve fine‐tuning of the endogenous GA_3_ content.

**Figure 4:**
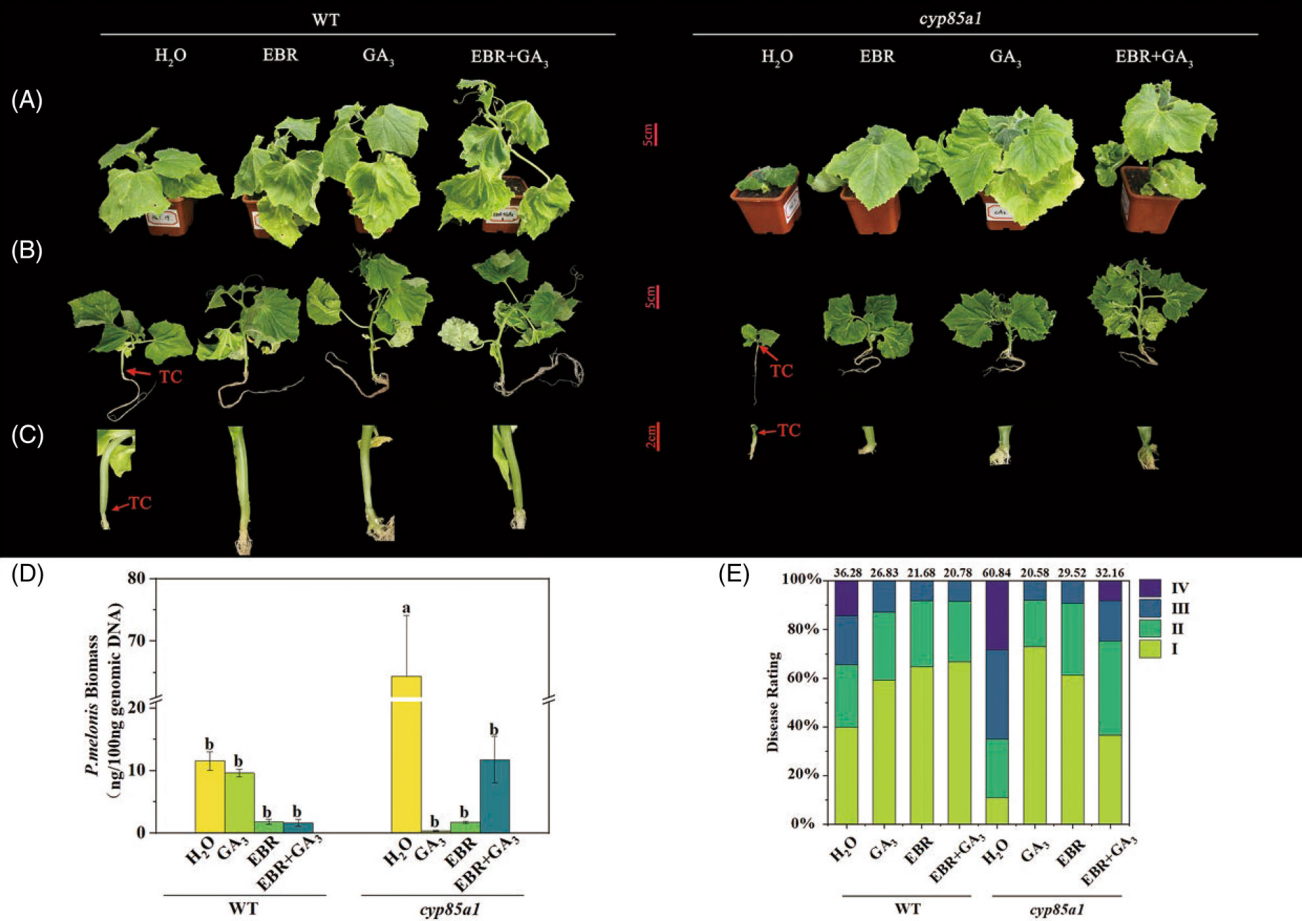
EBR and GA_3_ application suppressed *Phytophthora melonis*‐induced disease spreading on cucumber plants 3 days after inoculation with *P. melonis*. (A) Phenotypes of *cyp85a1* and WT plants with or without EBR and GA_3_ application on day 0 before inoculation. (B) Phenotypes of *P. melonis* infected *cyp85a1* and WT plants with or without EBR and GA_3_ application. (C) Closeup of visible water‐soaking, brownish lesions, or plant collapse of the hypocotyls. (D) *P. melonis* biomass assays. (E) Disease incidence assays. TC, tissue collapse. Data in (D) are shown as means ± SE of three biological replicates (*n* = 9). Different letters indicated significant differences (*P* < 0.05) according to Duncan's multiple range tests. EBR, 24‐epibrassinolide; WT, wild type.

To further verify the involvement of GAs in BRs‐regulated plant growth and disease resistance, we examined the effects of exogenous GA_3_ pretreatment. Complementing *cyp85a1* with exogenous GA_3_ completely rescued the hypocotyl diameter, leaf number, and leaf area; partly rescued petiole length; and further elongated the first internode length. However, hypocotyl lengthening was not promoted by GA_3_ treatment. Providing WT plants with exogenous GA_3_ also substantially promoted plant growth. Supplying plants with both GA_3_ and EBR exhibited additive effects in promoting the above growth parameters to different extents. Moreover, both the WT and *cyp85a1* mutant plants supplemented with exogenous GA_3_ or EBR had a lower chlorophyll content compared to that of the corresponding plants without chemical treatments. Notably, WT plants had no first internode (Figure 4A; Figure S6). In terms of disease resistance, exogenous GA_3_ treatment completely rescued the reduced resistance of the *cyp85a1* mutant to the level of WT plants, as indicated by a decreased in pathogen biomass accumulation and disease index. GA_3_ treatment also slightly increased resistance in WT plants. However, co‐treatment of EBR and GA_**3**_ did not confer an additive effect on the level of resistance in either the WT or *cyp85a1* mutant plants (Figure 4B–E).

**Figure 5:**
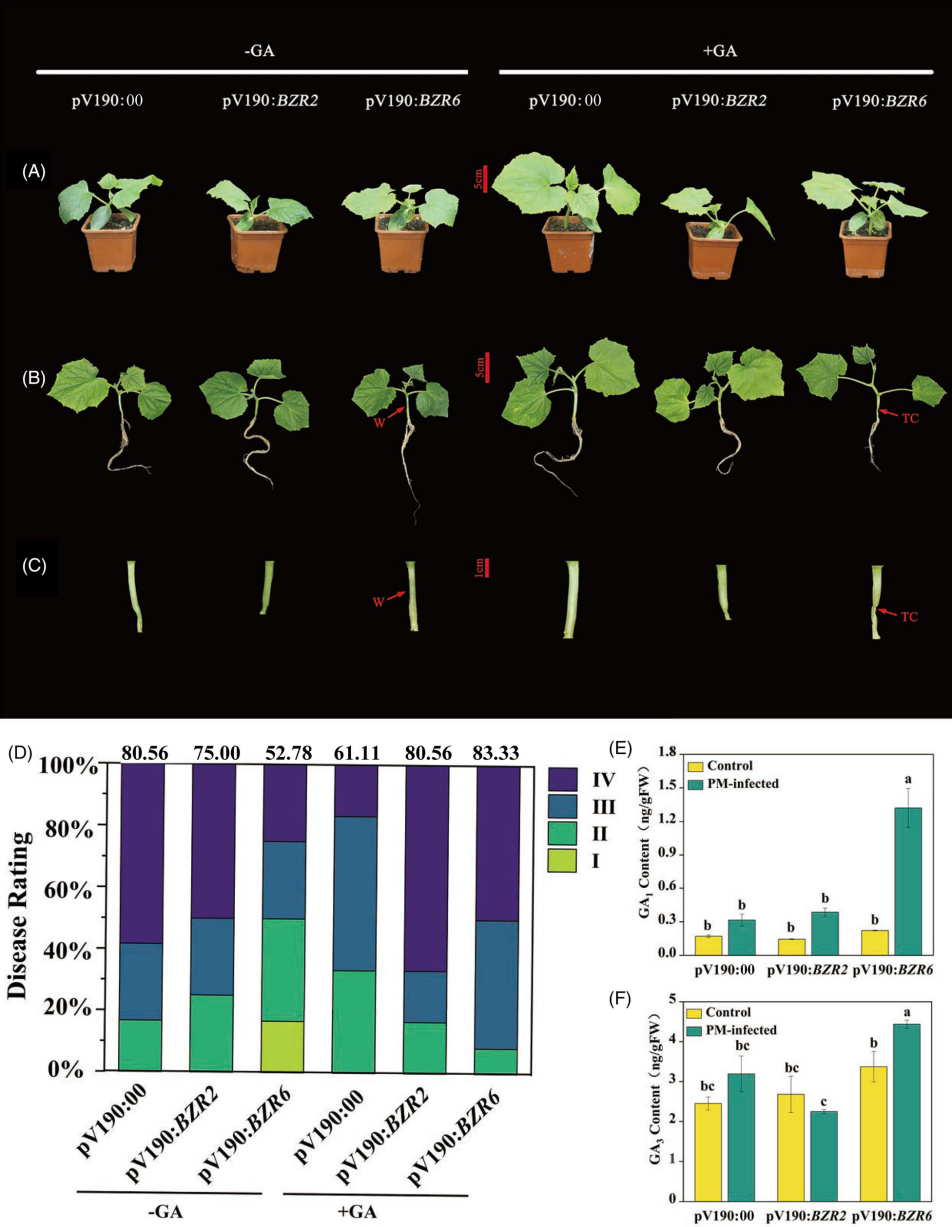
GA_3_ application abolished the disease resistance induced by silencing of *BZR6* 3 days after inoculation with *Phytophthora melonis*. (A) Phenotypes of *BZR2*‐ and *BZR6*‐silenced plants with or without GA_3_ application on day 0 before inoculation. (B) Phenotypes of *P. melonis* infected *BZR2*‐ and *BZR6*‐silenced plants with or without GA_3_ application. (C) Closeup of visible water‐soaking, brownish lesions, or plant collapse of the hypocotyls. (D) Disease incidence assays. (E) Endogenous GA_1_ levels. (F) Endogenous GA_3_ levels. TC, tissue collapse; W, water soaking with necrosis. Data in (E, F) are shown as means ± SE of three biological replicates (*n* = 9). Different letters indicated significant differences (*P* < 0.05) according to Duncan's multiple range tests.

To further investigate the role of BR signaling in plant growth and disease resistance, and to clarify the relationship between BRs and GAs in these processes, two homologous transcription factors mediating BR signaling (*BZR2* and *BZR6*) were silenced by VIGS. Transcript levels of *BZR2* or *BZR6* in the hypocotyls of plants infiltrated with pV190‐*BZR2* or pV190‐*BZR6* were confirmed to be 54% or 58% lower than those of plants infiltrated with the pV190 vector control, respectively (Figure S7). Silencing of *BZR2* or *BZR6* reduced hypocotyl thickness, but had no effect on the other growth indices; exogenous application of GA_3_ significantly increased the hypocotyl thickness, petiole length, and leaf number in *BZR2*‐ or *BZR6*‐silenced plants (Figure 5A; Figure S8). Moreover, silencing of *BZR6* prevented disease development, and the disease index decreased by 34.48% compared to that of plants infected with the control vector pV190. By contrast, silencing of *BZR2* had no effect on resistance. In response to *P. melonis* infection, both GA_1_ and GA_3_ accumulation in *BZR6*‐silenced plants increased to a greater extent than found in the pV190‐infected and *BZR2*‐silenced plants. In addition, pre‐treatment with GA_3_ abolished the enhanced resistance induced by silencing *BZR6*, with an increase in the disease index from 52.78% to 83.33% (Figure 5B–F). However, GA_3_ treatment had little effect on the disease resistance of *BZR2*‐ silenced plants.

### BRs regulate GA biosynthesis through the BZR6‐dependent transcriptional regulation of *GA20ox1*

**Figure 6:**
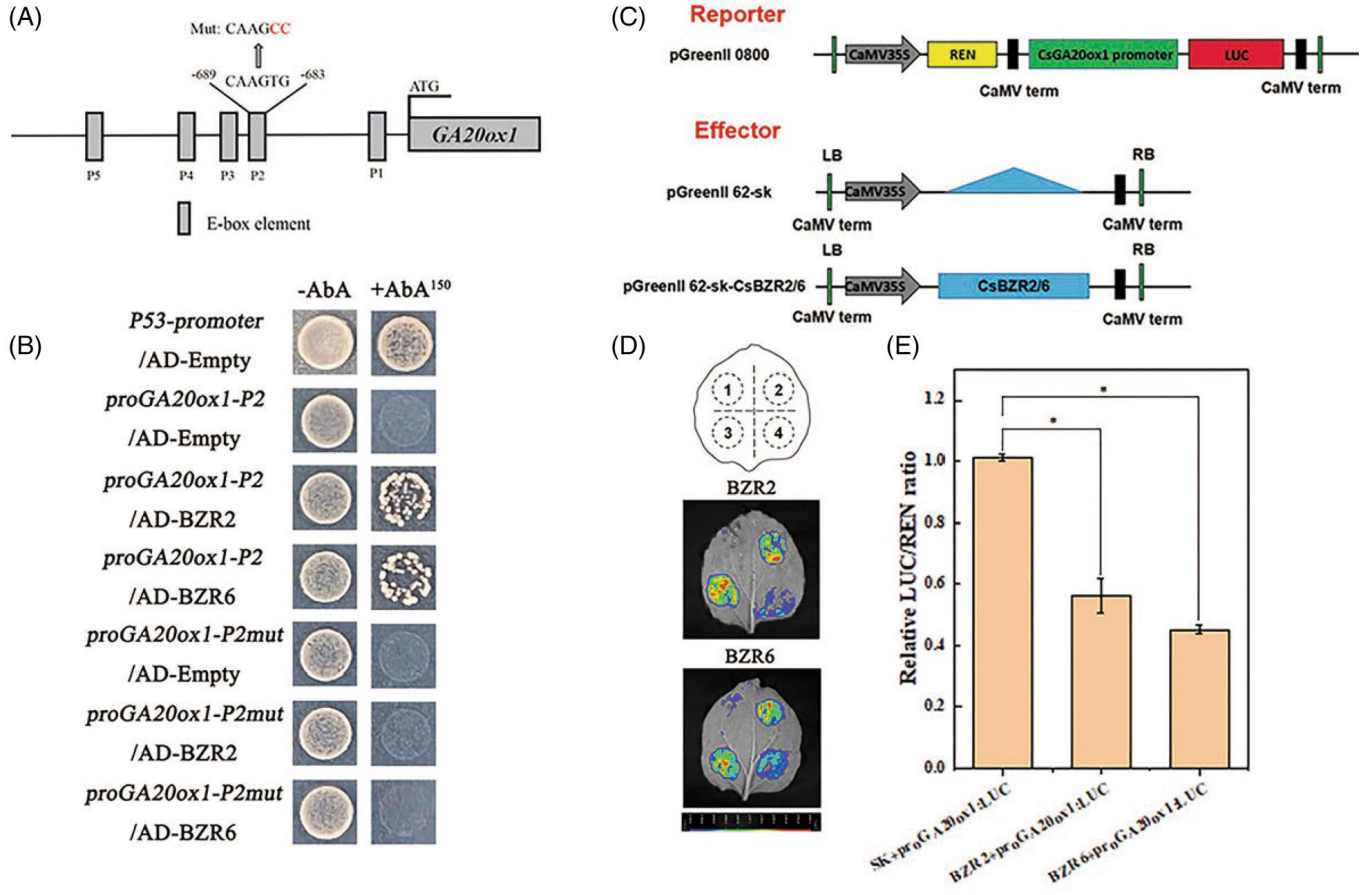
BZR2 and BZR6 directly suppressed the transcription of *GA20ox1*. (A) Schematic diagrams of the *GA20ox1* promoters. (B) Yeast one‐hybrid analysis of BZR2 or BZR6 binding to the *GA20ox1* promoter P2 region. WT and mutated promoter P2 region were used for analysis. Interaction was determined on synthetic defined (SD) medium lacking Leu in the presence of AbA (150 ng/mL). (C) Schematic diagram of the effectors, reporters, and internal compositions. (D) Dual luciferase reporter system assays showed the binding of BZR2 or BZR6 to the *GA20ox1* promoter in vivo. The fluorescence intensity weaker than control (empty vector) meant that BZR2 or BZR6 transrepressed the expression of *GA20ox1*. Label 1: Co‐expression of pGreenΙΙ 0800‐Luc vector and pGreenII 62‐SK vector. Label 2: Co‐expression of proGA20ox1::LUC and pGreenII 62‐SK vector. Label 3: Co‐expression of pGreenΙΙ 0800‐Luc vector and 35S::BZR2/6. Label 4: Co‐expression of proGA20ox1::LUC and 35S::BZR2/6. (E) Dual luciferase assay for the regulatory effect of BZR2 or BZR6 on the expression of *GA20ox1*. The ratio of LUC/REN of the SK plus promoter was set to 1 for normalization. Data in (E) are shown as means ± SE of three biological replicates (*n* = 9). Asterisks indicated significant differences (*P* < 0.05) according to Student's *t*‐test.

To verify whether *BZR2* or *BZR6* has a regulatory effect on the GA biosynthetic gene *GA20ox1*, the promoter region upstream of *GA20ox1* (upstream 1218 bp) was inspected. The promoter contains five E‐box elements (CANNTG), which could be a binding site of the BZR transcription factor (Figure 6A). The yeast‐one‐hybrid assay was performed using five different constructs, each containing one E‐box element. As shown in Figure 6(B) and Figure S9, only the yeast cells that contained the bait vector harboring the *GA20ox1* promoter P2 region grew normally on the deficient SD/‐Leu medium when transformed with BZR2‐AD or BZR6‐AD. In addition, the yeast cells harboring both the *GA20ox1pro*‐bait vector containing the mutated E‐box region of the *GA20ox1* promoter P2 and the pGADT7‐BZR2 or pGADT7‐BZR6 vector could not grow on the selective medium. A LUC assay was performed to further visualize the interaction. Examination of *N. benthamiana* infiltrated with the above constructs using a plant live imaging system showed that the fluorescence intensity was weaker than that of the control (empty vector), clearly demonstrating that BZR2 or BZR6 could bind to the *GA20ox1* promoter and consequently suppress *GA20ox1* expression (Figure 6C,D). The relative LUC activity assay also showed that the activity of the *GA20ox1* promoter was inhibited by 44% or 55% as a result of BZR2 or BZR6 binding, respectively (Figure 6E). Collectively, these results indicated that BZR2 and BZR6 negatively regulate *GA20ox1* transcription through direct binding to its promoter *in vitro*. For comparison, we also performed the yeast‐one‐hybrid assay for *GA20ox3*, which showed that neither *BZR2* nor *BZR6* interacts with *GA20ox3* (Figure S9).

### GA biosynthesis is necessary for BR‐mediated Phytophthora blight resistance in cucumber

**Figure 7:**
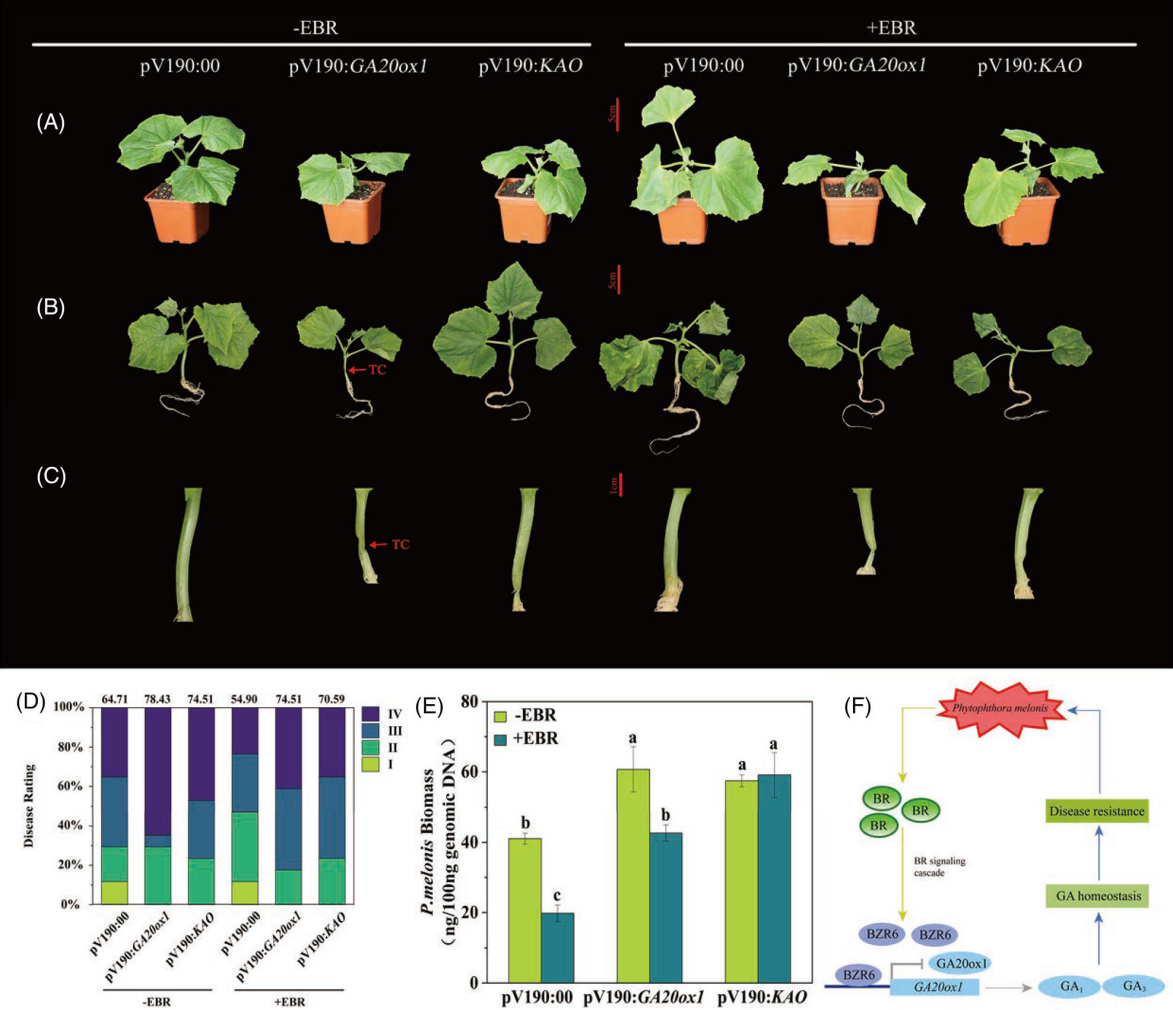
EBR‐induced disease resistance was blocked by silencing of *GA20ox1* or *KAO* 3 days after inoculation with *Phytophthora melonis*. (A) Phenotypes of *GA20ox1*‐ and *KAO*‐silenced plants with or without EBR application on day 0 before inoculation. (B) Phenotypes of *P. melonis* infected *GA20ox1*‐ and *KAO*‐silenced plants with or without EBR application. (C) Closeup of visible water‐soaking, brownish lesions, or plant collapse of the hypocotyls. (D) Disease incidence assays. (E) *P. melonis* biomass accumulation in the plants infected with *P. melonis*. TC, tissue collapse. (F) A proposed working model of BR‐GA crosstalk in the regulation of cucumber growth and *P. melonis* resistance. Data in (E) are shown as means ± SE of three biological replicates (*n* = 9). Different letters indicated significant differences (*P* < 0.05) according to Duncan's multiple range tests. BR, brassinosteroids; EBR, 24‐epibrassinolide.

To investigate whether GA biosynthesis is essential for the observed EBR‐enhanced plant growth and disease resistance, two GA biosynthesis genes, *GA20ox1* and *KAO*, were silenced by VIGS and then EBR was applied exogenously to the gene‐silenced plants for extensive analysis. The transcript levels of *GA20ox1* or *KAO* in the hypocotyls of plants infiltrated with pV190‐*GA20ox1* or pV190‐*KAO* were 82% or 57% lower than those of plants infiltrated with the pV190 vector control, respectively. Under non‐inoculation conditions, silencing of *GA20ox1* or *KAO* significantly inhibited plant growth; the plants exhibited delayed growth and dwarfism. The EBR‐induced petiole lengthening was hindered to different extents by silencing of *GA20ox1* or *KAO* (Figure 7A; Figure S10). Furthermore, the EBR‐induced disease resistance was blocked by silencing *GA20ox1* or *KAO*, as indicated by the increase in the disease index and *P. melonis* biomass. The repression of disease resistance was greater in *GA20ox1*‐silenced plants than in *KAO*‐silenced plants (Figure 7B–E).

## DISCUSSION

The continuous coevolutionary struggle of hosts and pathogens is complex. Plant hormones are small‐molecule signals, which not only orchestrate fundamental developmental cues but also convey environmental inputs and induce an adaptive response to abiotic and biotic stresses. Plant hormones, such as salicylic acid (SA), jasmonic acid (JA), and ethylene (ET), are well‐established to play an important role in plant immunity (Wang, Tyler et al., 2019). In this study, we found that GA biosynthesis is necessary for the BRs‐mediated increased resistance of cucumber plants to *P. melonis* and uncovered a regulatory module of BZR6‐GA20ox1.

Studies in plant–oomycete interactions elucidated the complex roles and action mechanisms of BRs in mediating the plant disease response using BR biosynthesis and signaling mutants along with exogenous treatment of active BRs (de Bruyne et al., 2014; Lozano‐Durán et al., 2013; Yu et al., 2018). Exogenous EBR treatment increased the resistance of soybean plants to *P. sojae* (Wang et al., 2020), and we also previously found similar results in cucumber plants against *P. melonis* (Ren et al., 2020). Silencing of the BR receptor gene *SERK3/BAK1* in potato increased the susceptibility of the plants to *P. infestans*, which was accompanied by reduced expression of the BR marker genes S*tSTDH*, *StEXP8*, *StCAB50*, and *StCHL1* (Wang, He et al., 2019). In tomato plants, the disease severity of *curl3*, a mutant that is insensitive to BR, was significantly higher than that of WT plants after inoculation with *P. infestans* (Dallagnol et al., 2023). In addition, overexpression of *MdBES1* or *MdMYB88* in apple increased the disease resistance against *V. mali* infection. *MdBES1* could directly bind to the *MdMYB88* promoter, and *MdMYB88* promoted BR biosynthesis by upregulating the expression of BR biosynthesis genes (Liu et al., 2021). In this study, both *P. melonis* inoculation of plants varying in BR biosynthesis capacity and exogenous application of EBR uncovered a positive role of BRs in the disease response (Figures 1 and 3), suggesting that BRs function as a resistance factor to enhance the resistance of cucumber plants to *P. melonis*. However, BZR6, a key transcription factor in the BR signaling pathway, was found to negatively regulate the resistance to *P. melonis* (Figure 5).

The mechanisms by which GA plays a role in regulating disease resistance are complex. The DELLA protein SLENDER RICE1 (SLR1) showed a resistance‐promoting effect against hemibiotroph rice pathogens by boosting SA‐ and JA‐mediated defense responses (de Vleesschauwer et al., 2016). Knockout of the *Elongated uppermost internode* (*Eui*) gene, encoding a GA deactivating enzyme, or exogenous application of GA_3_ reduced disease resistance to *Xanthomonas oryzae* pv. *oyrzae* and *Magnaporthe oryzae*, whereas *Eui* overexpression increased disease resistance (Yang et al., 2008). By contrast, inoculation of *GA20ox3*‐overexpression and RNA‐interference lines with the rice pathogens *M. oryzae* and *X. oryzae* pv. *oryzae* revealed a positive role of GA in disease resistance (Qin et al., 2013). Exogenous application of GA_3_ could restore the normal growth phenotypes of dwarf rice plants infected by *Rice dwarf virus* (Zhu et al., 2005). In addition, GA was found to mitigate the damage caused by citrus Huanglongbing (i.e., citrus greening disease) through inhibiting H_2_O_2_ production and cell death in phloem tissues (Ma et al., 2022). In the present study, knockdown of *KAO* or *GA20ox1* significantly reduced the resistance of cucumber to *P. melonis* (Figure 7). The effects of exogenously applied GA_3_ on the disease resistance of cucumber plants varied according to the genotype of the plant. In the *cyp85a1* mutant with a reduced GA_3_ level upon infection, GA_3_ application significantly enhanced resistance (Figures 3 and 4). However, for the WT, pV190‐infected, and *BZR2*‐silenced plants with no change in GA_3_ level upon infection, exogenous GA_3_ treatment only increased the resistance of WT and pV190‐infected plants, but had no influence on the resistance of *BZR2*‐silenced plants (Figures 4 and 5). Moreover, exogenous application of GA_3_ to *BZR6*‐silenced plants with a relatively increased endogenous GA_3_ level upon infection counteracted the resistance induced by silencing *BZR6* (Figure 5). Hence, we concluded that GA‐induced Phytophthora blight resistance in cucumber plants not only relies on modulation of the endogenous GA_3_ content, but also involves the interaction of GA with the BR signaling pathway. Consequently, GA_3_ over‐production might trigger feedback regulation of the BZR2/BZR6‐mediated resistance response.

Several lines of evidence indicate that BR/GA cross‐talk putatively plays a crucial role in the BR‐mediated resistance to *P*. *melonis*. First, transcriptional profiling of WT and mutant *cyp85a1* cucumber plants revealed the potential role of GA biosynthesis in BR‐mediated Phytophthora blight resistance (Figure 2; Figure S3). Second, functional analysis of CYP85A1, BZRs, GA20ox1, and KAO confirmed that GA biosynthesis is necessary for BRs‐induced disease resistance. The BR‐deficient *cyp85a1* mutant plants showed more severe disease symptoms than the WT plants, accompanied by a reduced endogenous GA_3_ content after pathogen infection (Figure 3F), whereas external foliar application of GA_3_ to the *cyp85a1* mutant completely rescued the resistance to the WT level (Figure 4). In addition, transient knock down of *BZR6* increased disease resistance and simultaneously promoted the accumulation of endogenous GA_1_ and GA_3_ in cucumber after *P. melonis* infection compared with those of WT plants (Figure 5E,F). More importantly, the disease resistance conferred by exogenous EBR was abolished by silencing *GA20ox1* or *KAO* (Figure 7). Third, the results of yeast one‐hybrid and luciferase assays suggested that BZR6 directly suppressed the transcription of *GA20ox1* through binding to its promoter (Figure 6). However, *CYP85A1*‐OE plants showed higher disease resistance than WT plants, but had lower GA_1_ and GA_3_ levels (Figure 3E,F), and the increased disease resistance induced by silencing *BZR6* was abolished by exogenous GA_3_ treatment (Figure 5). Co‐application of EBR and GA_3_ also did not have an additive effect on disease resistance compared to the application of either hormone alone in both WT and *cyp85a1* plants (Figure 4). Together, these results point to a complex interaction of BRs and GA_3_ in triggering the plant disease‐resistance response, suggesting that the fine‐tuning of endogenous GA content might be required for the BR‐induced resistance of cucumber plants to *P. melonis*. Nevertheless, our results are somewhat inconsistent with the findings of a previous study in rice showing that BR antagonized GA‐directed root immunity via indirectly stabilizing the DELLA protein and the central GA repressor SLR1 (de Vleesschauwer et al., 2012).

The ontogenic or age‐related resistance (ARR) phenomenon to Phytophthora fruit rot has been documented in Cucurbitaceae. The young fruits are extremely susceptible to *Phytophthora* infection but become resistant throughout growth and development (Alzohairy et al., 2020; Ando et al., 2009; Mansfeld et al., 2017, 2020). Although such an ARR pattern to Phytophthora root and crown rot has not been reported in Cucurbitaceae, it has been widely documented in Solanaceae vegetable crops including tomato (Shah et al., 2015), potato (Mutty & Hossenkhan, 2008) and pepper (Jeun & Hwang, 1991). In this study, the plants with reduced levels of endogenous BRs or GAs through knockout of the biosynthesis gene *CYP85A1*, *KAO*, or *GA20ox1*, resulting in delayed growth compared with that of WT plants, were also more susceptible to *P. melonis* (Figures 3 and 7; Figures S5 and S10). In addition, *CYP85A1* overexpression promoted plant growth and increased plant disease resistance (Figure 3; Figure S5). These results seem to support a mechanism of BR/GA‐mediated growth‐related resistance against *P. melonis* infection. However, no correlation between growth rate and disease resistance in *BZR6*‐ or *BZR2*‐silenced plants was observed, regardless of with or without GA_3_ treatment, which does not provide support for the ARR theory (Figure 5; Figure S8). We also did not find an additive promotion effect of EBR and GA_3_ on plant growth in WT or *cyp85a1* mutant plants with respect to pathogen resistance (Figure 4; Figure S6). Hence, a trade‐off between plant disease resistance and growth is required in the co‐evolutionary struggle of hosts and pathogens. BRs‐regulated disease resistance is not simply ascribed to a mechanism of growth‐related resistance, suggesting that the resistance mechanism is more complex.

## CONCLUSION

Our results provide evidence that BRs positively regulate the resistance of cucumber plants to the pathogen *P*. *melonis* and reveal a multifaceted regulation mechanism of BR/GA cooperation on growth‐defense trade‐offs. These findings can help to inform the development of a valuable strategy for cucumber blight control in production.

## EXPERIMENTAL PROCEDURES

### Plant materials

The wild‐type (WT) and *cyp85a1* mutant cucumber (*Cucumis sativus* L.) lines were kindly provided by Dr. Yuhong Li (Northwest A & F University). The WT line ‘Changchun mici’ (CCMC) is a North China fresh type inbred line, which was used for virus‐induced gene silencing (VIGS) and genetic transformation. The BR‐deficient mutant *cyp85a1* (encoding BR‐C6‐oxidase in the BR biosynthesis pathway) is an ethyl methanesulfonate (EMS)‐derived mutant isogenic CCMC cucumber line with lower BR levels relative to those of the WT. Loss of function of *CYP85A1* results in an extremely dwarf phenotype with practically no internodes, and small, dark green, and severely wrinkled leaves. Foliar spraying of 100 μM EBR partially rescues the mutant phenotypes (Wang et al., 2017).

### Treatments with exogenous plant hormones

We examined the effects of EBR and GA_3_ on the promotion/suppression of growth and disease resistance of *cyp85a1* mutant, WT CCMC plants, and WT plants with silencing of GA biosynthetic genes *GA20ox1* and *ent‐kaurenoic acid oxidase* (*KAO*) and BR signaling genes (*BZR2* and *BZR6*) individually.

The seedlings of *cyp85a1* mutant plants were sprayed with 5 mL of 100 μM EBR (Sigma‐Aldrich, Germany; catalog #E1641) and 5 mL of 100 μM GA_3_ (Solarbio, China; catalog #G8040) per plant every day when the cotyledons were fully expanded. Notably, the *cyp85a1* seedlings emerged from soil surface 1 week later than WT seedlings, due to the extremely short hypocotyl and practically no epicotyl in the mutant. To confirm restoration of the phenotype in *cyp85a1* via EBR administration, plant growth was evaluated 13 days after EBR treatments in WT and 6 days after treatment in *cyp85a1*, and then the plants were inoculated with *P. melonis* the following day. For evaluation of restoration/suppression of the phenotype in the *cyp85a1* by GA_3_, morphological traits and disease resistance were determined 15 days after GA_3_ treatments in WT plants and 8 days after treatment in *cyp85a1* plants, and then the plants were inoculated with *P. melonis* the following day.

The seedlings of *GA20ox1*‐ or *KAO*‐silenced plants at the two‐leaf stage were sprayed with 10 mL of 100 μM EBR per plant every day, whereas the seedlings of *BZR2*‐ or *BZR6*‐silenced plants at the two‐leaf stage were sprayed with 10 mL of 100 μM GA_3_ per plant every day. Plant growth was evaluated 5 days after chemical treatments, followed by inoculation with *P. melonis*. The stock solutions of EBR and GA_3_ were dissolved in ethanol and diluted with distilled water.

### Plant growth and inoculations

Cucumber plants were grown as described by Ren et al. (2020). In brief, the growth chamber (Ningbo, model RXZ 500‐D with fluorescent lamps) was programmed at 26°C during the day and 20°C at night under a 16‐h photoperiod with a 24 000‐lx light intensity, and the relative humidity was set to 70%. For the *cyp85a1* mutant plants treated with plant hormones, inoculation with *P. melonis* was initiated when the *cyp85a1* mutant plants developed two to three true leaves. For the *GA20ox1‐*, *KAO‐*, *BZR2‐* and *BZR6‐*silenced plants, *P. melonis* inoculation was initiated 5 days after plant hormones treatments. Considering that *cyp85a1* mutant plants present a dwarf hypocotyl phenotype, we supported the leaves from the soil with a 10‐μL pipette tip to avoid direct contact of the leaves with zoospores during inoculation (Figure S1).

A local isolate of *P. melonis*, TARIp211155 (accession no. MK386561.1) collected from cucumber in South China, was used for inoculations. Zoospore suspensions were prepared according to the method as described by Xu et al. (2015). The plants were inoculated by adding 20 mL of the zoospore suspensions (containing 1 × 10^4^ zoospores per milliliter) to the substrate near the stem base. Incubation was performed at 27°C in the dark for 24 h, followed by the normal diurnal cycle culture conditions mentioned above. Disease progression was observed 3 days after inoculation and scored based on a scale ranging from 0 to 4 as follows: 0, no symptoms; (1) barely water‐soaking or brownish lesions appearing on the crown; (2) water‐soaking or brownish lesions with slight constriction of the crown; (3) constrited/girdling crown progressed to become larger than 25% of the whole hypocotyl; and (4) death of the plant (Ren et al., 2020). The disease index (DI) was calculated from at least 10 individual plants per replicate according to the following equation:

$$\mathrm{DI}=\begin{array}{l} \frac{\sum \left(\text{disease scale value}*\text{number of plants}\;\mathrm{at}\;\text{each disease scale}\right)}{\text{maximum disease scale value}*\text{total number of plants surveyed}}\\ {}*100\% \end{array}$$

### *In vivo* analyses of *P. melonis* infection

For the visualization of fungal structures, the inoculated hypocotyl epidermis and transverse sections of inoculated hypocotyls were stained with lactophenol‐trypan blue as described previously (Tao, 2012) 3 days after inoculation. The transverse section of the hypocotyl (10 mm long starting from the basal end) was cut into small pieces with a razor blade.

### DNA extraction and fungal biomass quantification

DNA extraction and detection of *P. melonis* from cucumber hypocotyls were performed according to the methods of Wang et al. (2007). Briefly, fungal biomass accumulation was quantitatively assessed by amplifying the internal transcribed spacer region of the nuclear ribosomal DNA of *P. melonis* (Table S1). The calibration curve was constructed by plotting the logarithm of a known concentration (a 10‐fold dilution series from 100 ng to 10 pg in a reaction volume of 25 μL) of DNA from *P. melonis* against threshold cycle (*C*_t_) values. The regression equation was *C*_t_ = −3.6327 log[DNA] + 29.028, with a coefficient of determination (*R* ^2^) of 0.9997.

### RNA‐sequencing and differential gene expression analysis

When the seedlings developed two true leaves, the inoculated and non‐inoculated hypocotyls of *cyp85a1* mutant and WT plants were sampled for RNA‐sequencing analysis 3 days after *P. melonis* inoculation. RNA extraction and cDNA library construction of 12 cucumber hypocotyls were conducted by Biomarker Technologies (Beijing, China) according to standard procedures. Sequencing of all cDNA libraries was performed on an Illumina HiSeq2500 sequencer with approximately 50 million paired‐end strand‐specific reads produced per sample. Raw data (raw reads) in fastq format were processed through in‐house Perl scripts. Clean reads were obtained by removing reads containing adapters or poly‐N sequences and low‐quality reads from the raw data. A total of 117.28 Gb clean reads were generated, and the percentage of Q30 bases in each sample was greater than 94.06% (Table S2). Clean reads were aligned to the cucumber (Chinese Long) v3 reference genome using Hisat2 software and the mapped reads were quantified using StringTie software. Gene expression levels were estimated by the fragments per kilobase of transcript per million fragments mapped reads (FPKM) method. Differential expression analysis of two groups (Table S3; WT vs. *cyp85a1*; non‐inoculated vs. inoculated *cyp85a1*; non‐inoculated vs. inoculated WT; inoculated WT vs. inoculated *cyp85a1*) was performed using the DESeq2 package. Genes with a difference in expression level at an adjusted *P*‐value < 0.05 and fold Change ≥4 were considered to be differentially expressed genes (DEGs).

Gene Ontology (GO) enrichment analysis of the DEGs was implemented by the GOseq R packages based on the Wallenius non‐central hyper‐geometric distribution (Young et al., 2010). Venn Diagrams were generated using TBtools software (Chen et al., 2020). Enrichment analysis was performed using KOBAS (http://kobas.cbi.pku.edu.cn/genelist/) and the results were visualized with R tools (Bu et al., 2021).

### VIGS

Gene functions were analyzed using the VIGS system derived from the cucumber green mottle mosaic virus (Liu et al., 2020). Four target genes, *BZR2*, *BZR6*, *GA20ox1*, and *KAO*, were silenced using recombinant virus pV190 VIGS vectors as previously described (Liao et al., 2019) to analyze their functions during *P. melonis* infection in cucumber plants. The primers are listed in Table S1. In brief, the constructed plasmids were transformed into *Agrobacterium tumefaciens* strain GV3101. The VIGS inoculum was first infiltrated into the sprouts of the inbred cucumber line CCMC according to the methods of Liao et al. (2019), and then infiltrated into the cotyledons at the cotyledon stage to enhance the infection efficiency. Cucumber phytoene desaturase (PDS; encoded by *CsaV3_4G002690*) was used as a positive control to monitor the silencing efficiency based on the presence or absence of photo‐bleaching phenotypes. Plants infiltrated with an Agrobacterium culture carrying the empty pV190 VIGS vector served as controls. At the two‐leaf stage, the newly emerged leaves from the pV190‐*PDS*‐inoculated plants showed highly uniform photobleaching symptoms. The hypocotyls were sampled to quantify the silencing effect by determining the expression of targeted genes using reverse‐transcription quantitative polymerase chain reaction (RT‐qPCR). The plants that showed less than 50% transcript levels relative to those of control plants were foliarly applied with the plant hormone every day for 5 days, followed by inoculation with *P. melonis*.

### Cucumber transformation

To obtain cucumber plants overexpressing *CYP85A1* (*CYP85A1*‐OE), the full‐length coding sequence of *CYP85A1* was cloned into the pBI121‐GFP vector to generate the overexpression constructs. The *CYP85A1*‐OE constructs were introduced into *A. tumefaciens* via chemical transformation (Wise et al., 2006) and then transformed into the inbred cucumber line CCMC. Vacuum infiltration was used to enhance *Agrobacterium* infection. Green fluorescent protein (GFP) was used as a reporter in the selection of transgenic shoots (Nanasato et al., 2013; Zhang et al., 2017). GFP screening, PCR, sequencing, and western immunoblotting were used to characterize transgenic lines. The primers used for vector construction and transformation are listed in Table S1. Protein extraction and immunoblots analysis were performed according to the methods described by Yu et al. (2012). Total proteins isolated from the transgenic cucumber plants were assessed using an anti‐GFP tagged primary monoclonal antibody (Agrisera, Sweden; catalog #AS18 4175).

### Quantification of endogenous GA

For GA quantification, 1 g of the cucumber hypocotyl was sampled for each of three biological replicates. GA_1_ and GA_3_ levels were measured by ultra‐high‐performance liquid chromatography‐mass spectroscopy (Xevo TQD; Waters, Milford, MA, USA) coupled with an Alliance e2695 system (Waters) as described by Xin et al. (2020).

### Yeast one‐hybrid assay

The full‐length cDNA of *BZRs* was linked to the pGADT7 vector (Clontech‐Takara Bio) as a bait. The promoter fragment of *GA20ox1* and *GA20ox3* (containing the binding site of BZR1/BES1) was linked to the pAbAi vector as a prey. To obtain the mutant E‐box, the CAAGTG sequence was mutated to CAAGCC using the Mut Express MultiS Fast Mutagenesis Kit V2 (Vazyme, catalog # C215, Nanjing, China). The primers used to construct the vector are listed in Table S1. The pAbAi‐*GA20ox1* and *GA20ox3* construct were linearized and transferred into yeast cells and cultured on selective synthetic‐defined (SD) medium lacking uracil. Next, recipient cells containing *GA20ox1* and *GA20ox3* promoter fragments were prepared using the Yeastmaker™ Yeast Transformation System 2 (Clontech‐Takara Bio, catalog #630439, Japan), and *BZRs* were co‐transformed into recipient cells and grown on the SD/‐Leu medium (AbA Clontech‐Takara Bio, catalog #630466, Japan).

### Dual‐luciferase reporter assays

The 2000‐bp promoter fragment containing the *GA20ox1* binding site was cloned into the pGreenΙΙ 0800‐Luc vector (Biovector, Beijing, China) to generate the reporter (proGA20ox1::LUC). The coding sequence of *BZR2* or *BZR6* was cloned into the pGreenII 62‐SK vector (Biovector, Beijing, China) to generate the effector (35S::BZR2/6). The effector and reporter plasmids were then co‐injected into the leaves of *N. benthamiana*. At 48 h after inoculation, fluorescein potassium salt (Solarbio, catalog #IL2330, China) was uniformly applied to the abaxial surface of the leaf, and the fluorescence signal was detected 10 min later using a plant live imaging system (Berthold/NightSHADE LB985) (Gao et al., 2023). Luciferase activities were measured on a Luminoskan Ascent microplate luminometer (Thermo, Waltham, MA, USA) using the LUC gene test kit (Promega, Madison, WI, USA) (Hellens et al., 2005).

### RNA isolation and gene expression analysis

Total RNA from samples of the WT and mutant plant hypocotyls was extracted using an RNA extraction kit (Tiangen, catalog # DP360, Beijing, China) following the manufacturer's protocol. Subsequently, 1 μg of total RNA was reverse‐transcribed into cDNA using PrimerScript™ RT reagent kit with gDNA Eraser (Clontech‐Takara Bio, catalog # RR047A, Japan). qPCR was conducted on a CFX Connect Real‐Time PCR Detection System (Bio‐Rad, USA) with ChamQ SYBR qPCR Master Mix (Vazyme, catalog # Q341‐02, China). The primers are listed in Table S1. The expression levels of target genes were normalized against the level of the ubiquitin extension protein (*UBI‐ep*) gene (CsaV3_5G031430) as the reference and calculated using the 2^−ΔΔCT^ method (Wan et al., 2010).

### Statistical analysis

Data are presented as mean ± standard error (SE). Growth and disease rating determinations were performed with at least 10 individual plants per biological replicate. Determinations of the other parameters were performed with three independent tissue pools per biological replicate, each tissue pool consisting of three to six plants. All data were statistically analyzed with SAS software (SAS Institute Inc., Cary, NC, USA) using one‐way analysis of variance (ANOVA) and Duncan's multiple range test and assessed at the 0.05 level of significance.

## AUTHOR CONTRIBUTIONS

YK, XY and MZ conceived the original research plan; Material preparation, data collection and analysis were performed by ZJ, CM, XN and GP. The first draft of the manuscript was written by YK and all authors commented on previous versions of the manuscript. All authors read and approved the final manuscript.

## CONFLICT OF INTEREST STATEMENT

The authors declare that there is no conflict of interest regarding the publication of this article.

## Supporting information


**Figure S1.** Pipette tips were used to support plant leaves to prevent direct contamination of leaves by *Phytophthora melonis*.
**Figure S2.** (A) An upset plot showing all significantly differentially expressed genes (adjusted *P* < 0.05, fold change >4) and the intersecting datasets in the hypocotyls of WT versus *cyp85a1* plants 3 days after inoculation. (B) Principal component analysis of the transcriptome data. *cyp85a1* PM infected, *Phytophthora melonis*‐infected *cyp85a1*; WT PM infected, *P. melonis*‐infected WT; WT, non‐inoculated WT; *cyp85a1*, non‐inoculated *cyp85a1*.
**Figure S3.** Enriched functional subcategories of up‐ (A) and down‐regulated (B) transcripts (adjusted *P*‐value <0.05) in the hypocotyls of WT versus *cyp85a1* plants 3 days after inoculation. Circles' size represents the number of genes (log10) for each functional subcategory. Complete dataset in Table S4. Enrich factor = (differential expressed gene number in a pathway/total differential expressed gene number)/(gene number in a pathway in the database/total gene number in the database). *cyp85a1* PM infected, *Phytophthora melonis*‐infected *cyp85a1*; WT PM infected, *P. melonis*‐infected WT; WT, non‐inoculated WT; *cyp85a1*, non‐inoculated *cyp85a1*.
**Figure S4.** (A) Relative *CYP85A1* expression levels in different transgenic cucumber lines. (B) Immunoblot analysis to identify *CYP85A1* transgenic cucumber lines with an anti‐GFP antibody. Ponceau staining of Rubisco was used as the loading control. Data in (A) were shown as means ± SE of three biological replicates (*n* = 9). Different letters indicated significant differences (*P* < 0.05) according to Duncan's multiple range tests.
**Figure S5.** The growth characteristics of *cyp85a1*, WT and *CYP85A1*‐OE plants under the non‐inoculation condition with *Phytophthora melonis*. Data were shown as means ± SE of three biological replicates (*n* = 45). Different letters indicated significant differences (*P* < 0.05) according to Duncan's multiple range tests.
**Figure S6.** The effect of EBR and GA_3_ application on the growth of *cyp85a1* and WT plants under the non‐inoculation condition with *P. melonis*. Data were shown as means ± SE of three biological replicates (*n* = 45). Different letters indicated significant differences (*P* < 0.05) according to Duncan's multiple range tests.
**Figure S7.** The leaf bleaching phenotype was observed 14 (A) and 21 days (B) after cotyledons infiltration in pV190‐*PDS* plants. (C, D) Silencing efficiencies of individual genes through virus‐induced gene silencing (VIGS) in cucumber hypocotyls were determined on day 0 before hormone treatment through RT‐qPCR. Data were shown as means ± SE of three biological replicates (*n* = 9). Asterisks indicated significant differences (*P* < 0.05) according to Student's *t*‐test.
**Figure S8.** The effect of GA_3_ application on the growth of *BZR2*‐ and *BZR6*‐silenced plants under the non‐inoculation condition with *Phytophthora melonis*. Data were shown as means ± SE of three biological replicates (*n* = 45). Different letters indicated significant differences (*P* < 0.05) according to Duncan's multiple range tests.
**Figure S9.** Yeast one‐hybrid analysis of BZR2 or BZR6 binding to the *GA20ox1* and *GA20ox3* promoters. (A) Schematic diagrams of the *GA20ox1* promoters. (B) Schematic diagrams of the *GA20ox3* promoters. (C) BZR2 or BZR6 did not bind to *GA20ox1* promoter P1, P3, P4, P5 regions. (D) BZR2 or BZR6 did not bind to the promoters of *GA20ox3*. Interaction was determined on synthetic defined (SD) medium lacking Leu in the presence of AbA (150 ng/mL).
**Figure S10.** The effect of EBR application on the growth of *GA20ox1*‐ and *KAO*‐silenced plants under the non‐inoculation condition with *Phytophthora melonis*. Data were shown as means ± SE of three biological replicates (*n* = 30). Different letters indicated significant differences (*P* < 0.05) according to Duncan's multiple range tests.

**Table S1.** List of primers used in this study.
**Table S2.** Overview of sequencing quality and alignments. *cyp85a1* PM infected, *Phytophthora melonis*‐infected *cyp85a1*; WT PM infected, *P. melonis*‐infected WT; WT, non‐inoculated WT; *cyp85a1*, non‐inoculated *cyp85a1*.
**Table S3.** List of *Cucumis sativus* differentially expressed genes comparing WT and *cyp85a1* hypocotyls infected with or without *Phytophthora melonis*. *cyp85a1* PM infected, *P. melonis*‐infected *cyp85a1*; WT PM infected, *P. melonis*‐infected WT; WT, non‐inoculated WT; *cyp85a1*, non‐inoculated *cyp85a1*.
**Table S4.** Functional enrichment analysis of differentially expressed transcripts in response to *Phytophthora melonis* infection and *CYP85A1* mutation. Functional classification from Bu et al., Nucleic Acids Research 2021. *cyp85a1* PM infected, *P. melonis*‐infected *cyp85a1*; WT PM infected, *P. melonis*‐infected WT; WT, non‐inoculated WT; *cyp85a1*, non‐inoculated *cyp85a1*.
**Table S5.** List of differentially expressed genes involved in GA biosynthesis in the hypocotyls of WT versus *cyp85a1* plants 3 days after *Phytophthora melonis*. *cyp85a1* PM infected, *P. melonis*‐infected *cyp85a1*; WT PM infected, *P. melonis*‐infected WT; WT, non‐inoculated WT; *cyp85a1*, non‐inoculated *cyp85a1*.

## Data Availability

The raw RNA‐Seq reads have been deposited in the National Center for Biotechnology Information BioProject database (http://www.ncbi.nlm.nih.gov/bioproject) with ID PRJNA858502.
