# Supplementary material for: A multifaceted crosstalk between brassinosteroid and gibberellin regulates the resistance of cucumber to Phytophthora melonis
Source: Plant J. 2024 Jun 3;119(3):1353–68. doi: 10.1111/tpj.16855 (PMC13087484; doi:10.1111/tpj.16855)
Supplement: Supplementary file 1 — Figure S1. Pipette tips were used to support plant leaves to prevent direct contamination of leaves by Phytophthora melonis. Figure S2. (A) An upset plot showing all significantly differentially expressed genes (adjusted P < 0.05, fold change >4) and the intersecting datasets in the hypocotyls of WT versus cyp85a1 plants 3 days after inoculation. (B) Principal component analysis of the transcriptome data. cyp85a1 PM infected, Phytophthora melonis‐infected cyp85a1; WT PM infected, P. melonis‐infected WT; WT, non‐inoculated WT; cyp85a1, non‐inoculated cyp85a1. Figure S3. Enriched functional subcategories of up‐ (A) and down‐regulated (B) transcripts (adjusted P‐value <0.05) in the hypocotyls of WT versus cyp85a1 plants 3 days after inoculation. Circles' size represents the number of genes (log10) for each functional subcategory. Complete dataset in Table S4. Enrich factor = (differential expressed gene number in a pathway/total differential expressed gene number)/(gene number in a pathway in the database/total gene number in the database). cyp85a1 PM infected, Phytophthora melonis‐infected cyp85a1; WT PM infected, P. melonis‐infected WT; WT, non‐inoculated WT; cyp85a1, non‐inoculated cyp85a1. Figure S4. (A) Relative CYP85A1 expression levels in different transgenic cucumber lines. (B) Immunoblot analysis to identify CYP85A1 transgenic cucumber lines with an anti‐GFP antibody. Ponceau staining of Rubisco was used as the loading control. Data in (A) were shown as means ± SE of three biological replicates (n = 9). Different letters indicated significant differences (P < 0.05) according to Duncan's multiple range tests. Figure S5. The growth characteristics of cyp85a1, WT and CYP85A1‐OE plants under the non‐inoculation condition with Phytophthora melonis. Data were shown as means ± SE of three biological replicates (n = 45). Different letters indicated significant differences (P < 0.05) according to Duncan's multiple range tests. Figure S6. The effect of EBR and GA3 appli [file TPJ-119-1353-s002.pdf]

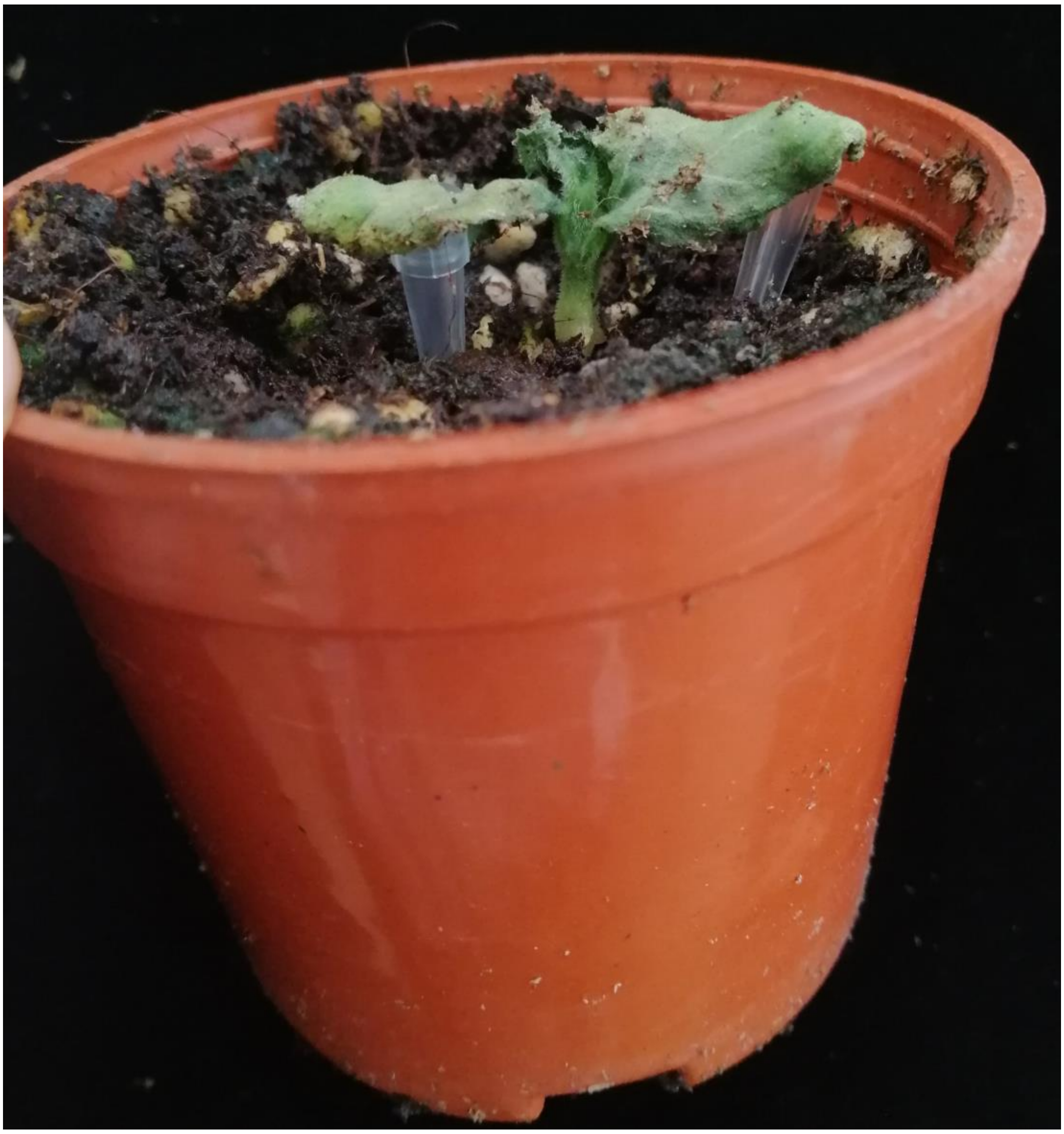

Figure S1 Pipette tips were used to support plant leaves to prevent direct contamination of leaves by *P. melonis*.

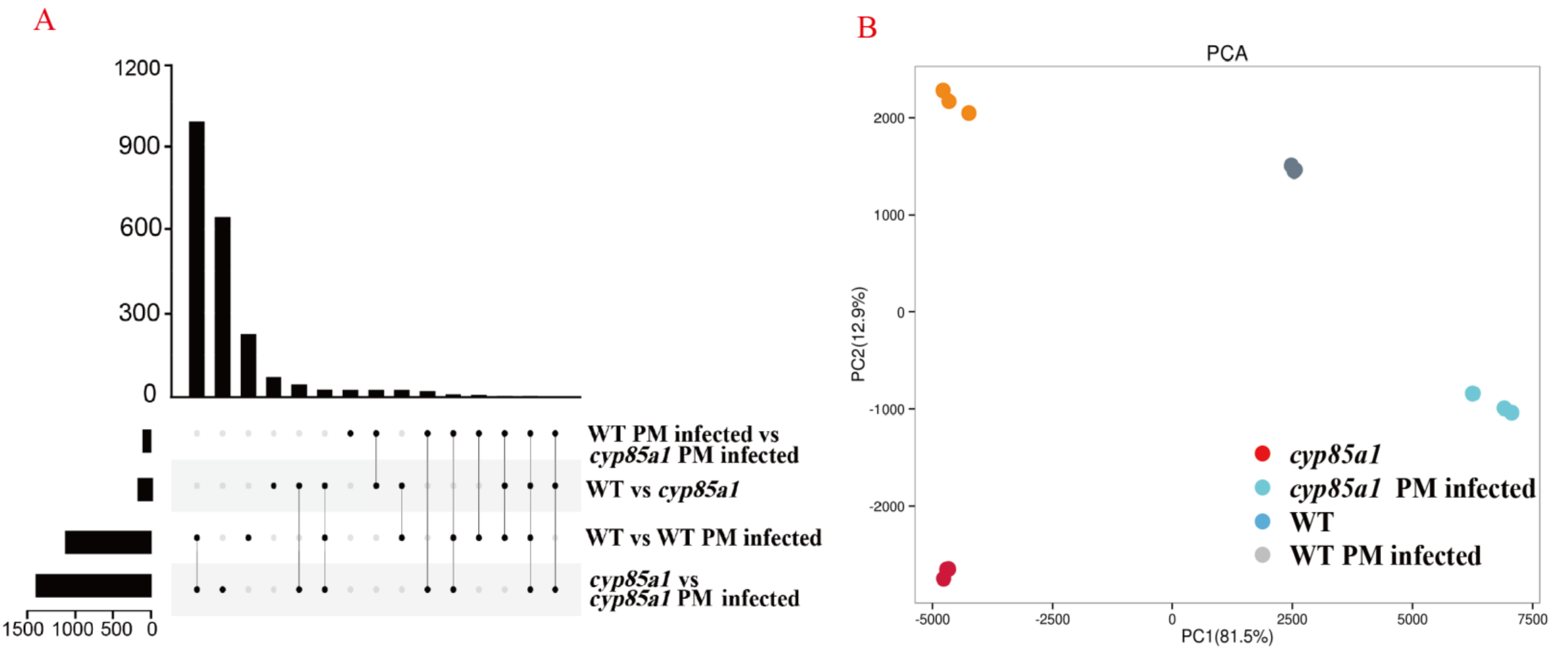

Figure S2 (A) An upset plot showing all significantly differentially expressed genes (adjusted P<0.05, fold change>4) and the intersecting datasets in the hypocotyls of WT vs *cyp85a1* plants on 3 days after inoculation. (B) Principal component analysis of the transcriptome data. *cyp85a1* PM infected, *P. melonis*-infected *cyp85a1*; WT PM infected, *P. melonis*-infected WT; WT, non-inoculated WT; *cyp85a1*, non-inoculated *cyp85a1*.

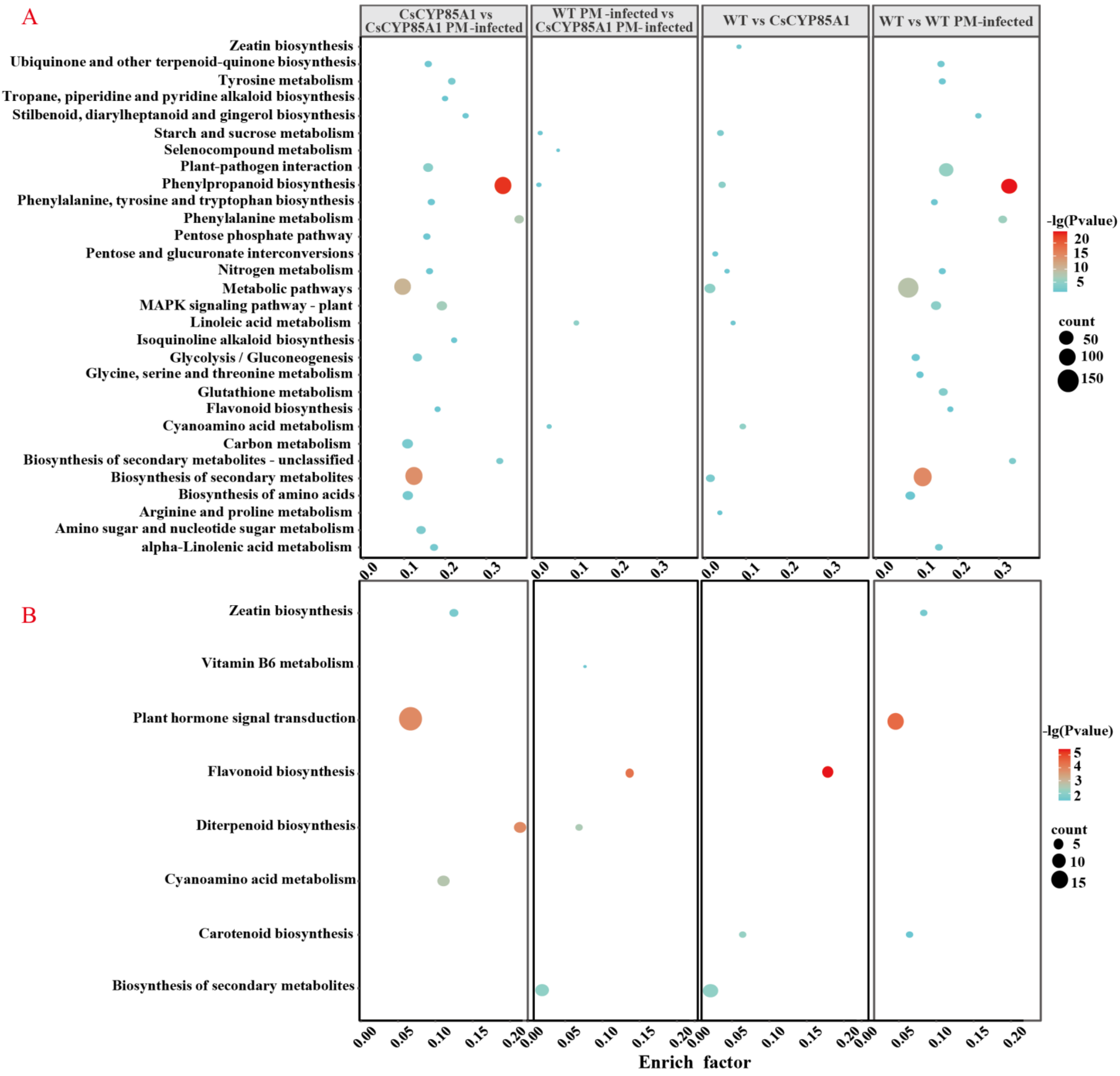

Figure S3 Enriched functional subcategories of up- (A) and down-regulated (B) transcripts (adjusted p-value <0.05) in the hypocotyls of WT vs *cyp85a1* plants on 3 days after inoculation. Circles' size represents the number of genes (log10) for each functional subcategory. Complete dataset in Supplementary Table S4. Enrich factor = (differential expressed gene number in a pathway / total differential expressed gene number)/(gene number in a pathway in the database/total gene number in the database). *cyp85a1* PM infected, *P. melonis*-infected *cyp85a1*; WT PM infected, *P. melonis*-infected WT; WT, non-inoculated WT; *cyp85a1*, non-inoculated *cyp85a1*.

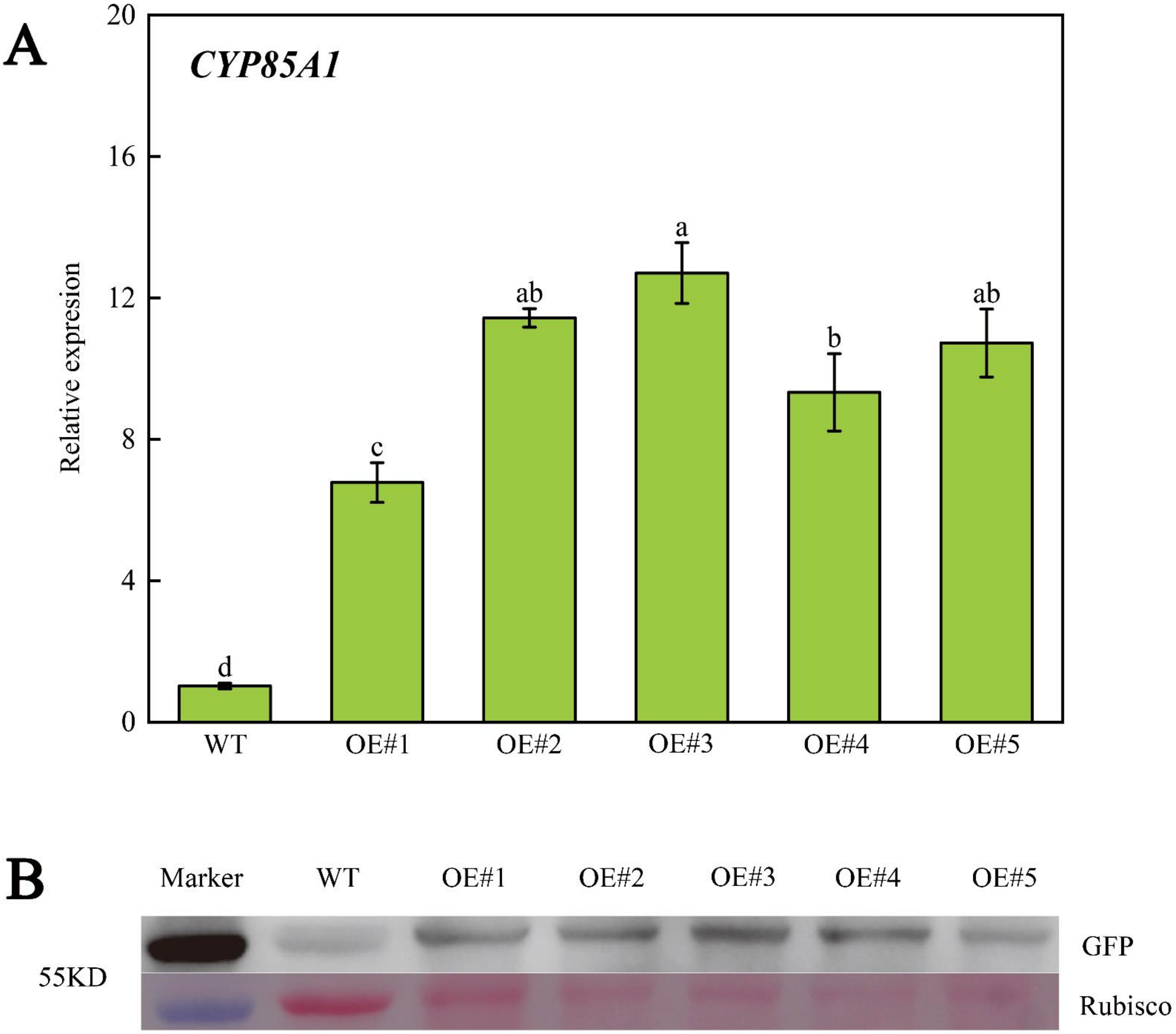

Figure S4 (A) Relative *CYP85A1* expression levels in different transgenic cucumber lines. (B) Immunoblot analysis to identify *CYP85A1* transgenic cucumber lines with an anti-GFP antibody. Ponceau staining of Rubisco was used as the loading control. Data in (A) were shown as means ± SE of three biological replicates (n=9). Different letters indicated significant differences ($P < 0.05$) according to Duncan's multiple range tests.

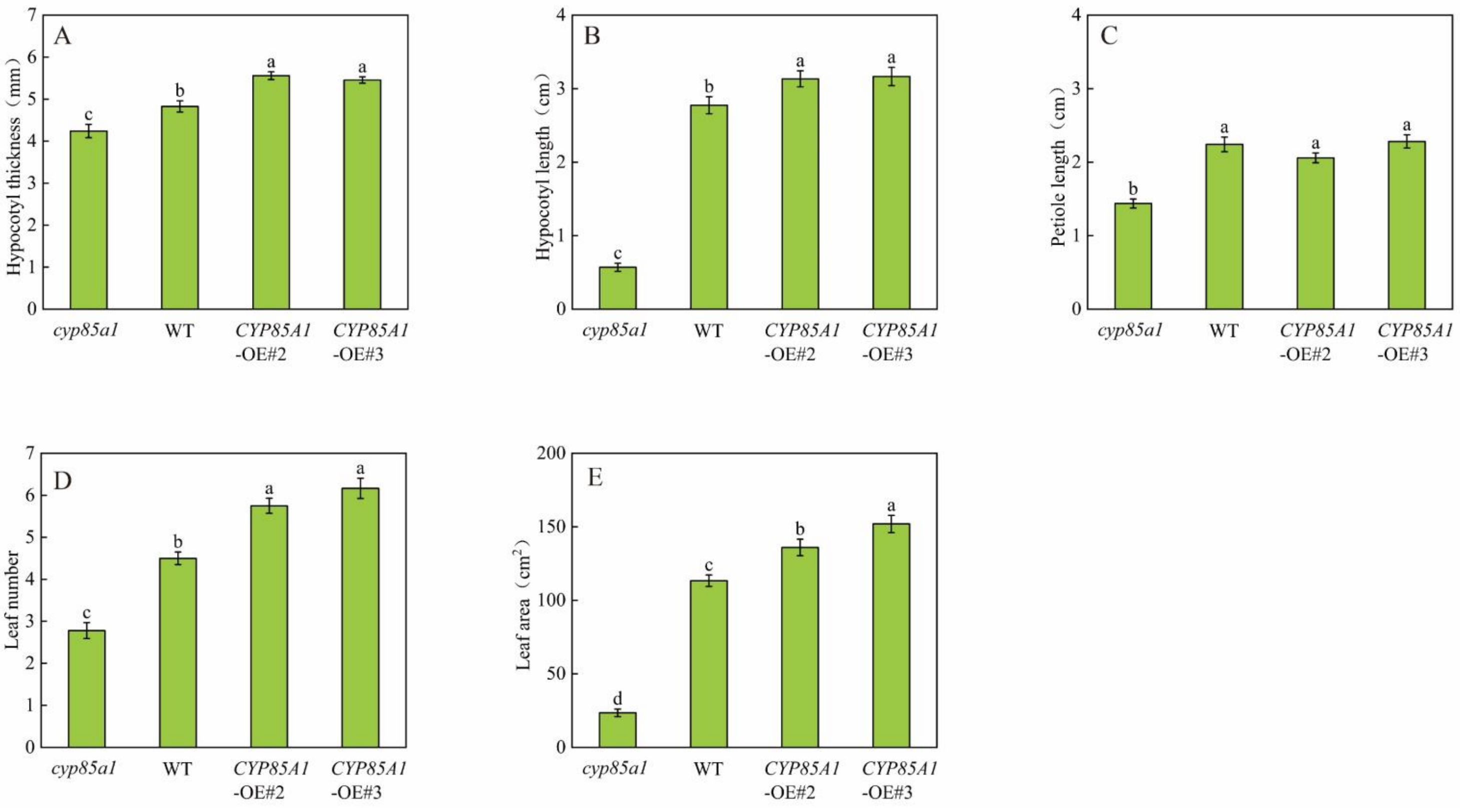

Figure S5 The growth characteristics of *cyp85a1*, WT and *CYP85A1*-OE plants under the non-inoculation condition with *P. melonis*. Data were shown as means ± SE of three biological replicates (n=45). Different letters indicated significant differences ($P < 0.05$) according to Duncan's multiple range tests.

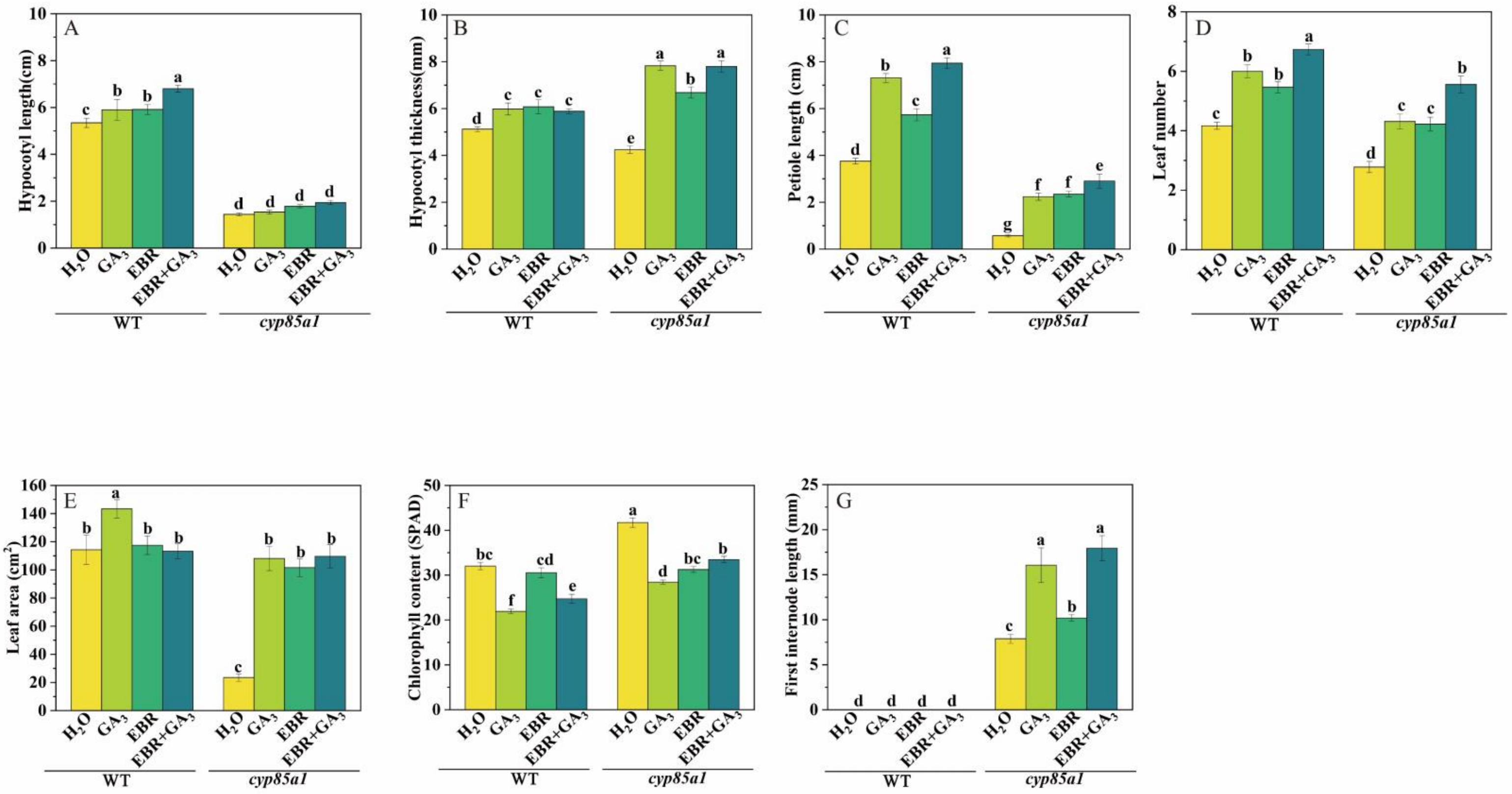

Figure S6 The effect of EBR and GA_3 application on the growth of *cyp85a1* and WT plants under the non-inoculation condition with *P. melonis*. Data were shown as means ± SE of three biological replicates (n=45). Different letters indicated significant differences ($P < 0.05$) according to Duncan's multiple range tests.

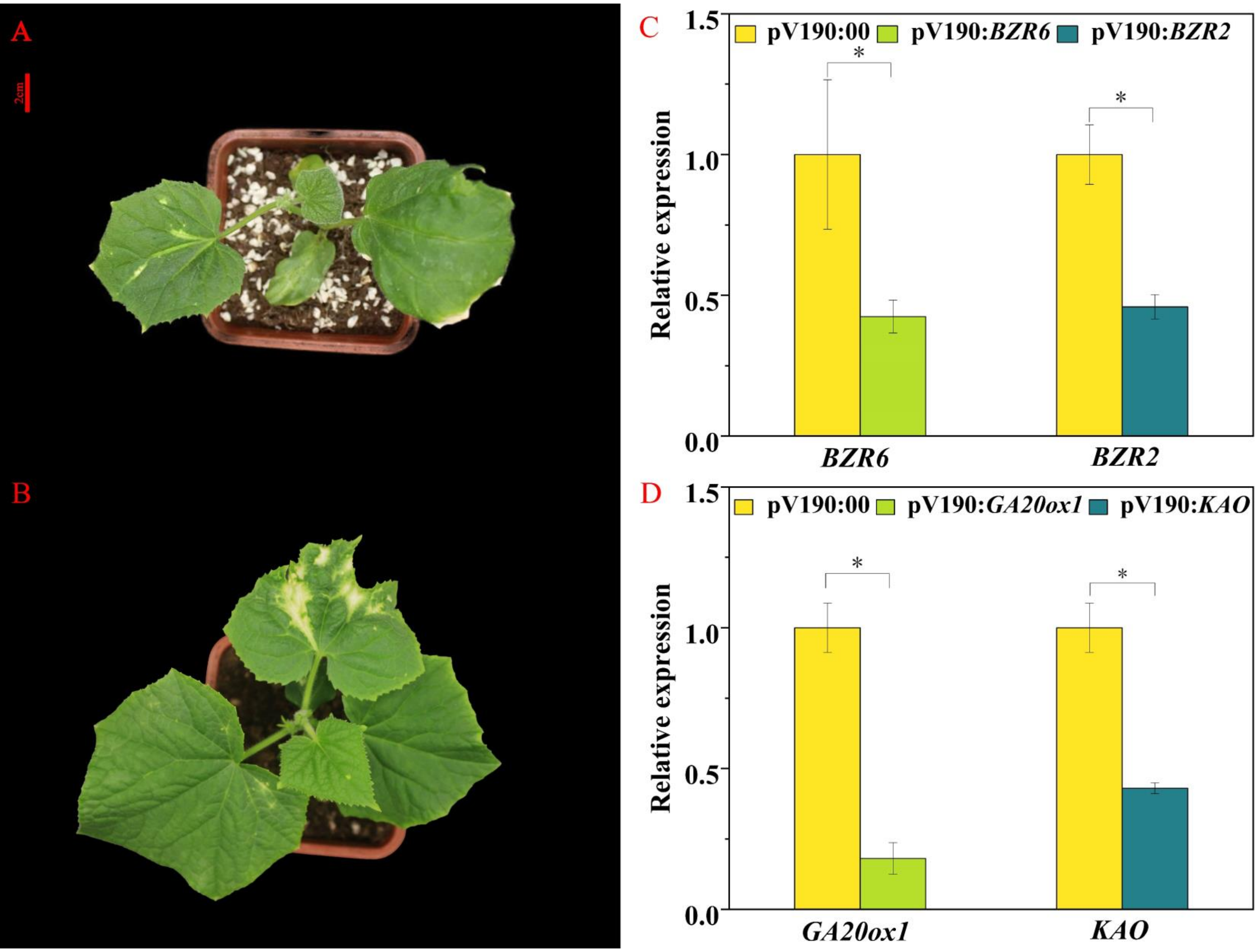

Figure S7 The leaf bleaching phenotype was observed 14 (A) and 21 days (B) after cotyledons infiltration in pV190-*PDS* plants. (C-D) Silencing efficiencies of individual genes through virus-induced gene silencing (VIGS) in cucumber hypocotyls were determined on day 0 before hormone treatment through RT-qPCR. Data were shown as means ± SE of three biological replicates (n=9). Asterisks indicated significant differences (P < 0.05) according to Duncan's multiple range tests.

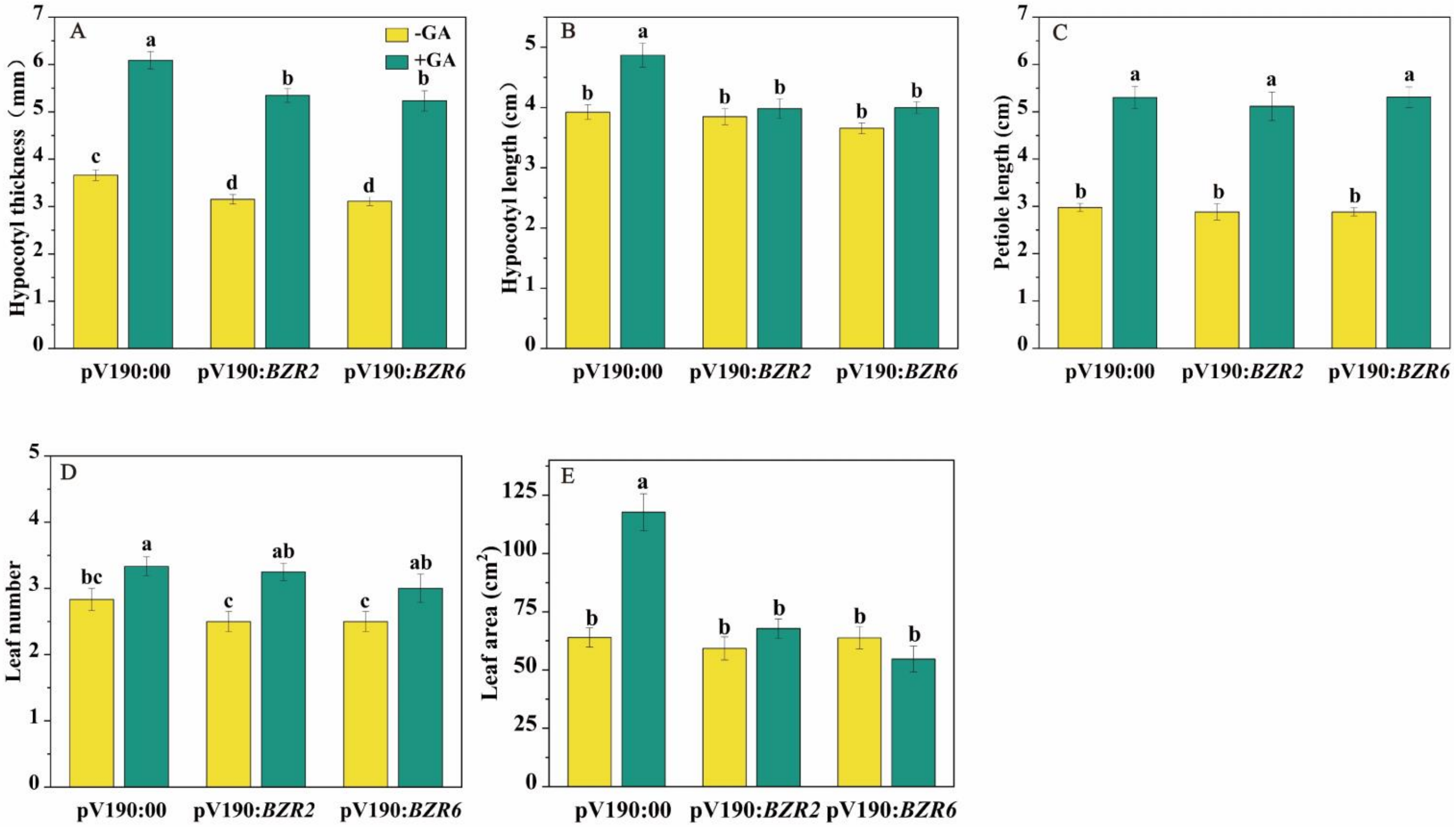

Figure S8 The effect of GA_3 application on the growth of *BZR2*- and *BZR6*-silenced plants under the non-inoculation condition with *P. melonis*. Data were shown as means ± SE of three biological replicates (n=45). Different letters indicated significant differences ($P < 0.05$) according to Duncan’s multiple range tests.

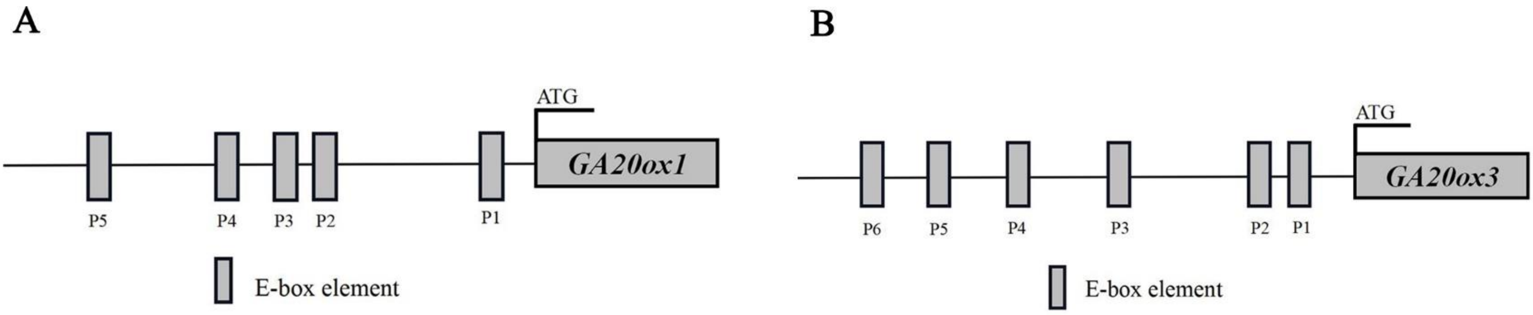

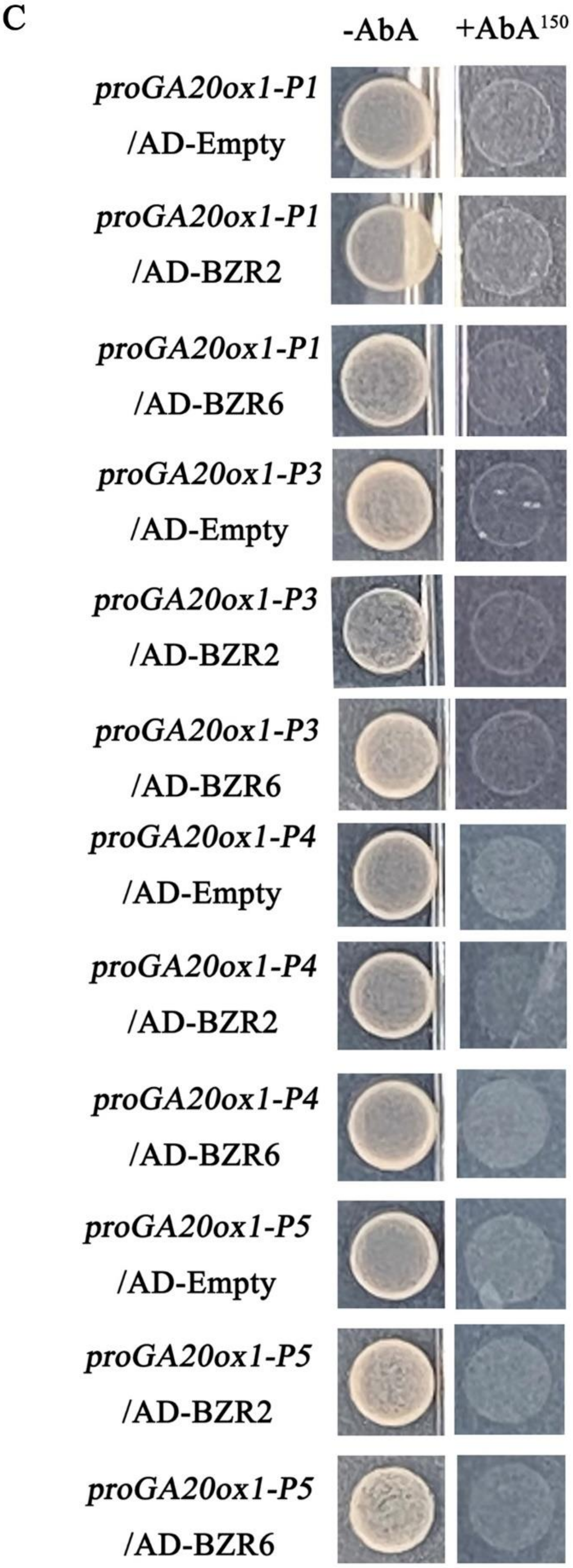

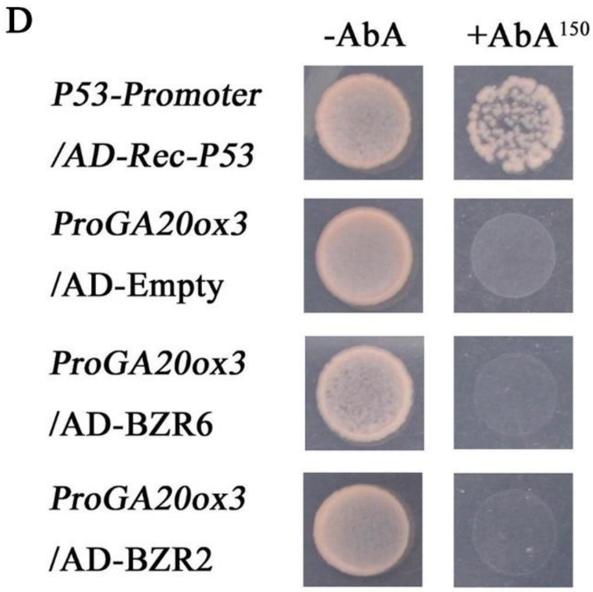

Figure S9 Yeast one-hybrid analysis of BZR2 or BZR6 binding to the *GA20ox1* and *GA20ox3* promoters. (A) Schematic diagrams of the *GA20ox1* promoters. (B) Schematic diagrams of the *GA20ox3* promoters. (C) BZR2 or BZR6 did not bind to *GA20ox1* promoter P1, P3, P4, P5 regions. (D) BZR2 or BZR6 did not bind to the promoters of *GA20ox3*. Interaction was determined on synthetic defined (SD) medium lacking Leu in the presence of AbA (150 ng/mL).

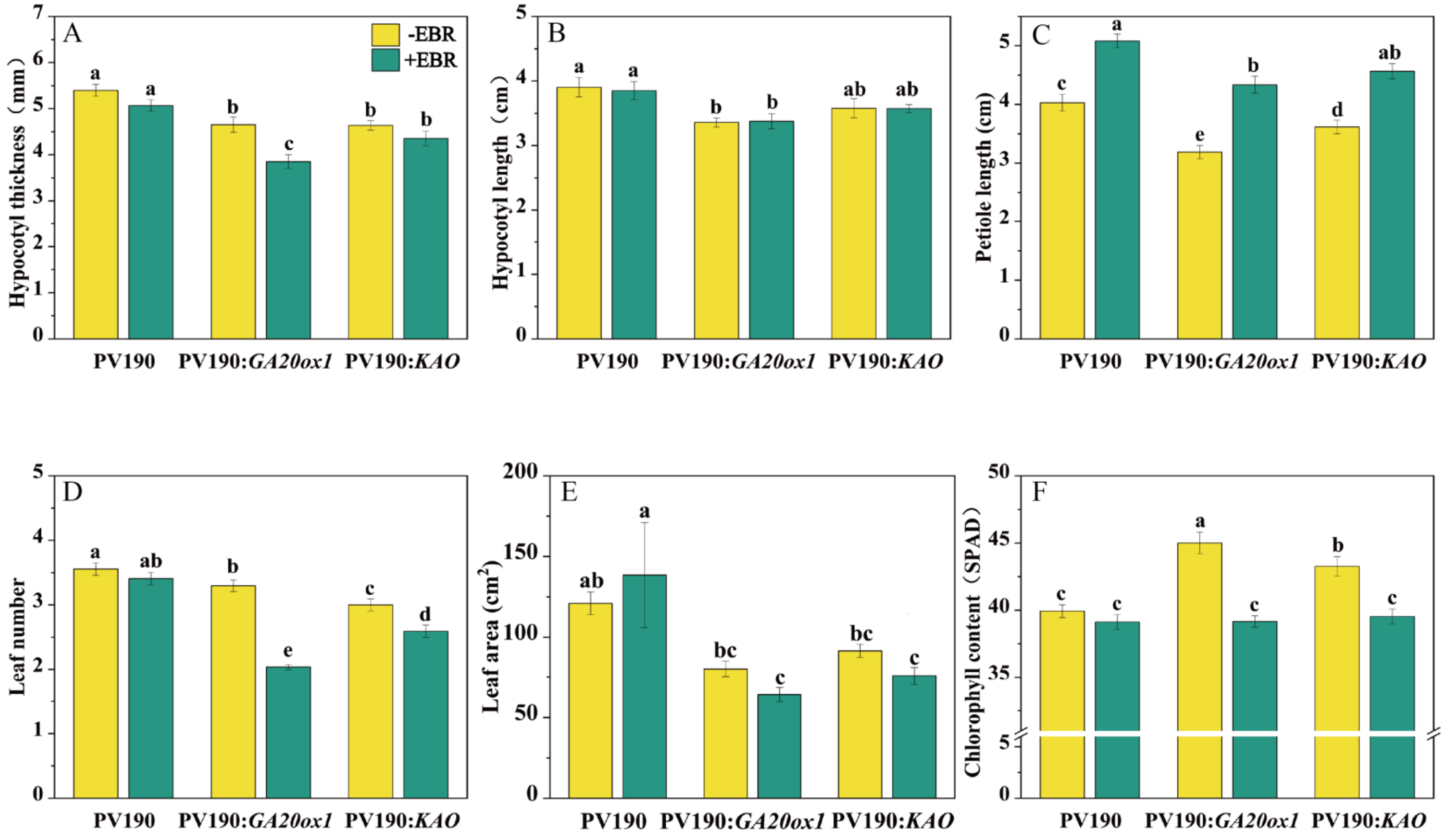

Figure S10 The effect of EBR application on the growth of *GA20ox1*- and *KAO*-silenced plants under the non-inoculation condition with *P. melonis*. Data were shown as means ± SE of three biological replicates (n=30). Different letters indicated significant differences (P < 0.05) according to Duncan’s multiple range tests.
